# Long‐acting injectable cabotegravir and rilpivirine in observational cohort studies: A systematic review on virological failure, resistance and re‐suppression outcomes in virally suppressed individuals living with HIV

**DOI:** 10.1111/hiv.70057

**Published:** 2025-06-13

**Authors:** Kyle Ring, Alexa Elias, Megan Devonald, Melanie Smuk, Chloe Orkin

**Affiliations:** ^1^ SHARE Collaborative, Blizard Institute, Faculty of Medicine and Dentistry Queen Mary University of London London UK; ^2^ Barts Health NHS Trust London UK

**Keywords:** cabotegravir rilpivirine, clinical practice, long‐acting ART, resistance, re‐suppression, virological failure

## Abstract

**Introduction:**

Randomized controlled trial evidence suggests that long‐acting injectable (LA‐I) cabotegravir and rilpivirine (CAB+RPV) has similar virological failure (VF) rates to daily oral therapy, but clinical practice evidence is lacking. Integrase inhibitor (INI) resistance may limit future therapy. The optimal regimen is uncertain.

**Methods:**

We synthesized evidence from PubMed, EMBASE, Cochrane and conference abstract databases through 18 November 2024, to identify observational cohort studies (OCS) that reported on VF events in virally suppressed individuals who switched to LA‐I CAB+RPV. We extracted data on VF, resistance‐associated mutations (RAMs) at VF, post‐VF regimen choice and re‐suppression. We assessed the risk of bias using a modified Downs and Black tool.

**Results:**

VF definitions differed considerably among OCS, with 172 individuals experiencing VF across 79 cohorts that included 13 899 individuals. Twenty‐eight cohorts (*n* = 7987) reported genotypic information at VF. Out of the 80 VF events with genotypic information available at the time of the VF event, NNRTI mutations were identified in 45 cases, INIs in 40 cases, and dual‐class resistance in 33 cases. Notably, 28 VF events were not accompanied by resistance. Post‐VF regimen choices were reported for 92 VF events. Regimens used were protease inhibitor (PI)‐based, oral INI‐based and some physicians continued LA‐I CAB+RPV post‐VF. Re‐suppression occurred in 87.8% (65/74) of VF events in which it was described.

**Conclusions:**

In OCS, VF was a very uncommon occurrence and comparable with clinical trials. However, when it did occur, it was frequently accompanied by resistance. Post‐VF regimens used to achieve suppression varied, including LA‐I CAB+RPV maintenance and were highly successful.

## INTRODUCTION

Long‐acting injectable (LA‐I) cabotegravir and rilpivirine (CAB+RPV) is recommended for people with HIV‐1 who are virally suppressed, with caveats around prior resistance and hepatitis B infection in international guidelines (where licensed and available) [1]. In specific instances, it is also suggested for non‐virally suppressed individuals who cannot take oral ART and are at risk of opportunistic infections [2, 3]. The virological failure (VF) rate across five randomized trials and three implementation studies ranges from 0% to 2.4% [4, 5, 6, 7, 8, 9, 10, 11]. While VF rates are similar to modern daily integrase inhibitor (INI)‐based oral therapy, acquiring one‐ or two‐class drug resistance is more likely, particularly when combined with two factors such as: BMI ≥ 30, A6 sub‐type or baseline RPV resistance‐associated mutations (RAMs) [12]. Since the roll‐out of LA‐I CAB+RPV for virally suppressed individuals, single‐site, multisite national, and multicountry observational cohort studies (OCS), case‐controlled cohorts, claims‐based studies and case series cohorts have been presented and published [13, 14, 15, 16, 17, 18, 19, 20]. These OCS vary significantly in terms of their size. Only three of 79 OCS in this review include more than 1000 individuals, while five include 500 to 1000 individuals. Furthermore, 43 OCS describe less than 100 individuals.

Most cohorts concentrate on clinical outcomes such as VF, with a smaller number of cohorts also reporting on the emergence of resistance, discontinuations, reasons for discontinuation and regimen use post‐VF [14, 15, 16, 17]. The evidence quality is variable (mostly low to moderate), and the median follow‐up duration for OCS is heterogeneous and short, typically ranging from 6 to 12 months.

Across international clinical guidelines in general, definitions of VF vary. However, in contrast with oral therapy, VF definitions in the context of LA‐I CAB+RPV are more stringent and incorporate new elements within the definition of VF, such as discontinuation [13, 14, 15, 16, 17, 18, 19, 20, 21]. For instance, a French cohort described VF as an unconfirmed VL > 200 c/mL or 2 VLs > 50 c/mL making comparisons between studies difficult [21]. The OPERA cohort and other large clinical cohorts have defined VF as ‘2 VLs ≥ 200 c/mL or 1 VL ≥ 200 c/mL + discontinuation’. [13] A large multi‐country Delphi process suggests that this disparity in definition may be due to lack of confidence in the genetic barrier of LA‐I CAB+RPV therapy [22]. Smaller cohorts describing non‐virally suppressed individuals have commenced reporting clinical outcomes, thereby emphasizing the need for a comprehensive understanding of the evidence in routine care for virally suppressed individuals [23, 24].

Consequently, ongoing evidence synthesis of clinical outcomes such as VF, emergence of resistance and re‐suppression is warranted. Perez Navarro et al. conducted an early meta‐analysis that concluded on 15 May 2024, and included both randomized controlled trials (RCTs) and OCS in individuals receiving LA‐I CAB+RPV [25]. The primary outcome was emergence of major INI RAMs, based on the Stanford Algorithm measured as a proportion of the total number of successfully genotyped participants with VF. NNRTI and dual‐class resistance were not reported.

INI resistance was reported in 2635 individuals across 19 studies, with a prevalence of 61% at the time of VF. Notably, seven of these studies were RCTs. The secondary outcome was the proportion of participants who received at least one injection and in whom VF occurred. VF prevalence was categorized into three clinical groups: induction maintenance, virally suppressed and non‐virally suppressed cohorts, with rates of 1%, 1% and 5%, respectively. The meta‐analysis included 33 studies with VF endpoints, of which 29 were conducted in virally suppressed individuals; seven of these studies were RCTs, and 22 were OCS. The follow‐up periods for the cohorts were heterogeneous and short, but the seven RCT study durations ranged from 48 to 124 or 152 weeks, resulting in an overall median follow‐up period of 41 months. VF definitions were also variable, contributing to significant heterogeneity. We present a larger and more clinically focused systematic review that exclusively focuses on OCS in virally suppressed populations. We categorize RAMs into INI, NNRTI and dual‐class resistance categories and provide detailed summaries of baseline/historic resistance and resistance at VF. Furthermore, we provide a summary of post‐VF ART regimen choices and re‐suppression rates, which enhances the evidence base and will guide clinical practice.

## MATERIALS AND METHODS

The OUTCOMES Study is a two‐year evidence synthesis project based on an ongoing systematic literature review. Its objective is to summarize OCS evidence from both published and grey literature regarding virological outcomes in individuals with HIV who transition to LA‐I CAB+RPV in various clinical contexts (namely virally suppressed and non‐suppressed populations). This manuscript pertains to Phase 1 of the OUTCOMES Study, which focuses on virological outcomes of LA‐I CAB+RPV in populations that are virally suppressed at the time of the switch.

### Search strategy and protocol

This systematic review followed the Preferred Reporting Items for Systematic Reviews and Meta‐Analysis (PRISMA) guidelines [26]. The protocol for the OUTCOMES Study was approved on 10 June 2024 https://doi.org/10.17605/OSF.IO/V68UN.

A systematic review to identify all OCS reporting virological outcomes in people with HIV who switched to LA‐I CAB+RPV was conducted. The search was first performed on 21 July 2024, and then updated on 28 November 2024, covering published and grey literature released between 1 January 2020, and 28 November 2024 [27, 28]. A supplementary search strategy was developed and adapted for use in Embase, PubMed and Cochrane (Tables S1–S3), and 23 HIV‐related congresses were hand searched (Table S4). The authors were contacted for clarification of results.

### Study screening and inclusion criteria

Multiple stages of screening were conducted. Preliminary screening of reports relevant to the OUTCOMES Study was carried out by two independent reviewers (discrepancies resolved by a third reviewer). Search results were uploaded into Covidence Systematic Review Software, de‐duplicated, and then screened using titles and abstracts, then full text (where applicable). During preliminary screening, outputs which did not describe OCS (e.g., literature reviews, studies with experimental designs), or which did not evaluate LA‐I CAB+RPV treatment for HIV‐1 were excluded.

Secondary screening was performed by members of the research team (A.E. with consultation by C.O.) in accordance with a predefined set of inclusion criteria organized using the PICOS framework specifically tailored for Phase 1 of the OUTCOMES Study (Table S5). During the screening conducted by the research team, studies were excluded if they included fewer than 10 individuals at the switch to LA‐I CAB+RPV, or if they did not provide data on virological outcomes (e.g., viral load or clinical endpoints at follow‐up). Additionally, studies that did not report on whether or not individuals were virally suppressed at the switch, or that did not provide discrete virological outcomes for virally suppressed individuals were excluded.

When a study yielded multiple research outputs, we prioritized the one with the most recent data. Manuscripts were given preference over congress materials (where available and up to date). Efforts were made to minimize overlap, where feasible. In the event of substantial overlap between multiple outputs, the output with the larger sample was selected.

### Data extraction and quality assessment

Following initial extraction using Microsoft Excel against the broader OUTCOMES Study, the study team (A.E. and cross‐checked by C.O.) performed extraction for studies that met the inclusion criteria for this review. Specific data fields are presented in Table S6.

The risk of bias of included studies was assessed using criteria from the Downs and Black checklist [29]. Similar to previous reviews that utilized this tool to appraise OCS and studies without an experimental study design, the checklist was modified to be applicable to the included studies. The modified tool is available in Table S7.

### Outcomes and data analysis

We have summarized the total number of individuals included across OCS who were at risk of VF and/or resistance in each OCS.

We have summarized the number of VF events observed, and, where available, included genotypic data on RAM presence at VF and at baseline (and/or historical RAMs), post‐VF regimens and re‐suppressions post‐VF, descriptively. We have categorized follow‐up durations into six‐month periods. We have classified post‐VF ART regimens by third agent as: oral INI, protease inhibitor (PI), non‐nucleoside reverse transcriptase inhibitor (NNRTI), multi‐tablet regimens (regimens composed of more than one ‘third‐agent’), LA‐I CAB+RPV, and therapeutic gap. We have reported the proportion with re‐suppression, where known. This is available in Table S9.

We have described virological failure events by individual in each study. We have recorded a VF definition for each cohort where described. Where a VF definition was not explicitly defined, we have used another marker of VF (such as resistance or VL exceeding the specified VL cut‐off, such as VL 200 c/mL) mentioned in the abstract and recorded this as the VF definition. We have labelled the definition as not reported if the study stated the presence or absence of VF but did not define it.

#### Role of the funding source

No writing support was provided. Inizio Medical was funded to perform the literature search and primary screening as outlined in the protocol.

## RESULTS

### Literature search

**FIGURE 1:**
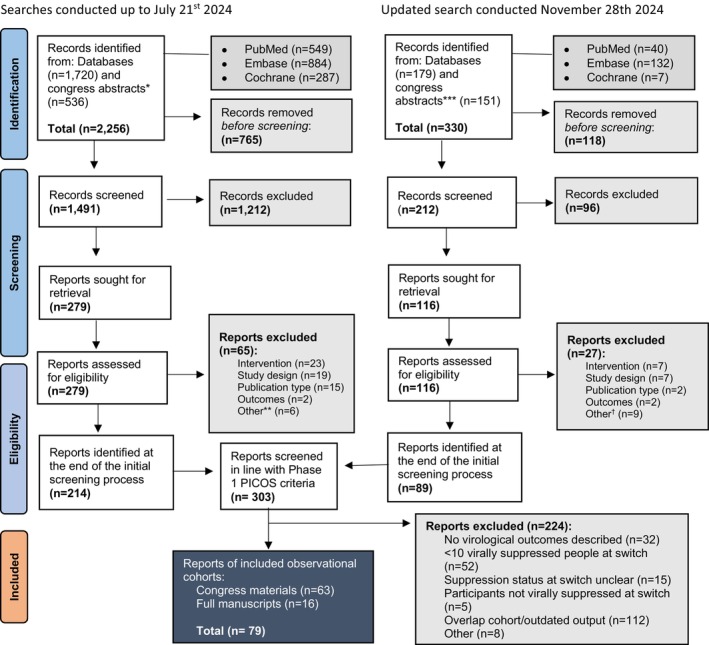
PRISMA Flow Diagram of Search Results. Searches conducted up to 21 July 2024. Updated search conducted on 28 November 2024. *American College of Clinical Pharmacy (ACCP) 2020–2022; American Conference for the Treatment of HIV (ACTHIV) 2022; AFRAVIH 2020–2024; AIDS 2020–2024; Australasian HIV and AIDS Conference (ASHM AHAC) 2021–2022; British Association for Sexual Health and HIV (BASHH) 2024; British HIV Association (BHIVA) 2019–2024; BHIVA/BASHH 2021; Canadian Conference on HIV/AIDS Research (CAHR) 2019–2024; Conference on Retroviruses and Opportunistic Infections (CROI) 2018–2024; European AIDS Clinical Society (EACS) 2023; European Meeting on HIV and Hepatitis (EMHH) 2023–2024; Grupo de Estudio del SIDA‐SEIMC (GeSIDA) 2018–2023; HIV Glasgow 2018–2022; HIV and Hepatitis in the Americas (HIV/HEP) 2018–2019; International AIDS Society/International AIDS Conference (IAS/IAC) 2019–2023; International Conference on Antiviral Research (ICAR) 2021; Italian Conference on AIDS and Antiviral Research (ICAR) 2021–2024; IDWeek 2019–2023; International Workshop on HIV Drug Resistance and Treatment Strategies 2023; The Professional Society for Health Economics and Outcomes Research (ISPOR) 2021–2024; ISPOR EU 2019–2022; Japanese Society for AIDS Research (JSAR) 2020–2021. **The number of individuals treated with CAB+RPV LA was not clear (*n* = 1); not available and encore included instead (*n* = 1); only one of 16 PLWH received CAB+RPV LA. Data for this participant were not separately reported (*n* = 1); data for PLWH treated with CAB+RPV LA were not reported in the abstract (*n* = 1); lack of information for the individuals treated with CAB+RPV LA (*n* = 1); subject in the study died from other complications soon after starting CAB+RPV LA treatment (*n* = 1). ***All congresses revisited to ensure the most complete abstract book/source material available searched. Additional results were found from: AFRAVIH 2020 and 2022; AIDS 2020; European AIDS Clinical Society (EACS) 2023; European Meeting on HIV and Hepatitis (EMHH) 2020–2021; Grupo de Estudio del SIDA‐SEIMC (GeSIDA) 2022; Société Française de Lutte Contre le Sida (SFLS) 2022; Japanese Society for AIDS Research (JSAR) 2020–2023. New searches: Australasian HIV and AIDS Conference (ASHM AHAC) 2024; HIV Glasgow 2018–2024; IDWeek 2024. IDWeek 2024 not fully searched by 28 November 2024 as *Open Forum Infectious Diseases* abstract book had not been published. ^†^Lack of information for the individuals treated with CAB+RPV LA (*n* = 3); no number of PLWH on CAB+RPV LA provided (*n* = 1); data on treatment with CAB+RPV LA were not separately reported (*n* = 4); abstract with incomplete data; data from final presentation captured (*n* = 1).

An ongoing literature search for the OUTCOMES Study initially identified 2256 records (Figure 1). After de‐duplication, titles and abstracts from 1491 records were screened. The remaining 279 were screened using full texts. Eligible records from a separate updated search (*n* = 89) were then added to the remaining initial 214 records. A combined total of 303 records identified from both searches were then screened again using a focused set of inclusion criteria, resulting in 79 included OCS (63 congress materials, 16 manuscripts) evaluating the use of LA‐I CAB+RPV in individuals who are virally suppressed at the time of the switch.

The 79 OCS included 13 899 virally suppressed individuals in whom 172 VF events occurred (Table 1). Of these, 28 cohorts reported genotypic data at VF in 7987 individuals (Table 2). Regarding re‐suppression, 25 cohorts reported on post‐VF regimens used and 23 reported on re‐suppression outcomes (Table 3). Of the 67 OCS that specified the dosing frequency, 75% (50/67) prescribed LA‐I CAB+RPV 2‐monthly and 25% (17/67) prescribed it both monthly and 2‐monthly (Table 1).

**TABLE 1:** Study characteristics and summary of virally suppressed individuals initiating LA‐I CAB+RPV in observational cohort studies.^a^

Study characteristics								VF
Study	Study country/countries	Follow‐up time, months^b^	Dose	VF definition	Virally suppressed at baseline, *N*	Female, *n (n/N%)* ^c^	Virally suppressed at risk of VF, *n* ^d^	VF, *n* (%)^e^
Studies with >1000 virally suppressed participants at baseline (*n* = 3)								
1. Hsu et al., 2024 (OPERA)	USA	NR	Q1M and Q2M	2 VL ≥ 200 c/mL or VL ≥ 200 c/mL + discontinuation	1362	237 (17.4)	1293	25 (1.9)
2. Martín et al., 2024 (RELATIVITY)	Spain	13	Q2M	NR	1285	183 (14.3)	1265	6 (0.5)
3. Deschanvres et al., 2023 (Dat'AIDS)	France	6.3 (median)	Q2M	2 VL > 50 c/mL or VL > 200 c/mL	1134	237 (20.9)	1134	14 (1.2)
Studies with 500–1000 virally suppressed participants at baseline (*n* = 5)								
4. Canavesi et al., 2024	Italy	5.23 (mean)	Q2M	NR	758	NR	758	14 (1.8)
5. Jongen, 2023 (Dutch ATHENA)	The Netherlands	9.6 (median)	Q2M	VL > 200 c/mL	619	61 (9.9)	588	5 (0.9)
6. Ring et al., 2024 (SHARE LAI‐net)	UK	7.5 (median)	Q2M	2 VL ≥ 200 c/mL	518	150 (28.9)	433	3 (0.7)
7. Muccini et al., 2024 (SCohoLART)	Italy	13.1 (median)	Q2M	2 VL ≥ 50 c/mL or VL ≥ 1000 c/mL	514	47 (9.1)	514	4 (0.8)
8. Gagliardini et al., 2024 (Icona)	Italy	NR	Q2M	2 VL > 50 c/mL or VL > 1000 c/mL + ART change	506	57 (11.3)	506	2 (0.4)
Studies with 100–500 virally suppressed participants at baseline (*n* = 28)								
9. Pozniak et al., 2024 (COMBINE‐2)	Switzerland, Germany, France, Spain, The Netherlands	3 (median)	Q2M	2 VL ≥ 200 c/mL or VL ≥ 200c/mL + discontinuation	472	53 (11.2)	374	3 (0.8)
10. Maguire et al., 2024	USA	8.2 (median)	Q1M and Q2M	2 VL ≥ 200 c/mL	374	94 (25.1)	374	3 (0.8)
11. Taramasso et al., 2024 (SCOLTA)	Italy	10 (median)	Q2M	NR	370	90/377 (23.9)^f^	370	4 (1.1)
12. Hessamfar et al., 2024 (ANRS CO3 AquiVIH‐NA)	France	12	Q2M	VL > 1000 c/mL or 2 VL > 50 c/mL	362	98/374 (26.2)^f^	362	9 (2.5)
13. Jonsson‐Oldenbüttel et al., 2024 (CARLOS)	Germany	12	Q2M	2 VL ≥ 200 c/mL or VL ≥ 200 c/mL + discontinuation	351	19 (5.4)	351	5 (1.4)
14. González‐Cordón et al., 2024	Spain	7	Q2M	2 VL > 50 cp/mL	313	25/318 (7.9)^f^	313	2 (0.6)
15. Palacios et al., 2024 (CARIPLA)	Spain	7.8 (median)	Q2M	VL ≥ 200 c/mL	281	36 (12.8)	264	7 (2.7)
16. Eron et al., 2024 (Trio Health)	USA	10 (median)	Q1M and Q2M	2 VL ≥ 200 c/mL or 1 VL≥200 c/mL+ discontinuation within 4 months of last injection	278	47/268 (17.5)^f^	221	2 (0.9)
17. Seang et al., 2023	France	12	Q2M	2 VL > 200 c/mL	270	44/283 (15.5)^f^	137	1 (0.7)
18. Ramirez, 2023 (Swiss HIV Cohort)	Switzerland	14 (median)	Q2M	2 VL > 50 c/mL	264	48 (18.2)	264	1 (0.4)
19. Hill et al., 2025	USA	14.5 (median)	Q1M and Q2M	2 VL > 200 c/mL	252	43/287 (15.0)^f^	252	2 (0.8)
20. Liu et al., 2024	USA	NR	NR	VL > 200 c/mL	233	132 (56.7)	53	1 (1.9)
21. Schneider et al., 2024 (BEYOND)	USA	12	Q1M and Q2M	2 VL ≥ 200 c/mL or VL ≥ 200 c/mL + discontinuation within 3 months	233	28 (12)	206	2 (1.0)
22. Hidalgo‐Tenorio et al., 2024 (CABO‐CHANCE)	Spain	7	Q2M	NR	224	22 (9.8)	149	0 (0)
23. Gutiérrez et al., 2024	Spain	11.1 (median)	Q2M	2 VL ≥ 200 c/mL or VL ≥ 1000 c/mL	173	25 (14.5)	173	2 (1.2)
24. Ferrara, 2024 (LARES)	Italy	11	Q2M	VL > 50 c/mL	172	27/176 (15.3)^f^	42	0 (0)
25. Dawiec et al., 2024	Poland	24	Q2M	NR	170	NR	170	2 (1.2)
26. An Chiu et al., 2024	USA	6	NR	VL > 50 c/mL	169	29 (17.2)	128	5 (3.9)
27. Fessler et al., 2024	USA	NR	Q1M and Q2M	VL ≥ 200 c/mL	153	NR	141	6 (4.3)
28. Psomas et al., 2023	France	18	Q2M	NR	142	31 (21.8)	142	1 (0.7)
29. Matone et al., 2024	Italy	10.75 (median)	Q2M	2 VL > 50 c/mL 4 weeks apart or VL > 1000 c/mL	138	32 (23.2)	138	0 (0)
30. Fernández‐Hinojal et al., 2023	Spain	4.1 (median)	Q2M	VL ≥ 50 c/mL	135	17/139 (43.6)^f^	128	3 (2.3)
31. Lagi et al., 2024 (LAHIV)	Italy	7 (median)	Q2M	2 VL > 50 c/mL or 1 VL > 1000 c/mL + ART change	129	23 (17.8)	129	2 (1.6)
32. Serris et al., 2024	France	10.5 (median)	Q2M	VL > 200 c/mL+ discontinuation, or 2 VL > 50 c/mL	126	33 (26.2)	126	5 (4)
33. Ribera et al., 2023	Spain	5	Q2M	NR	124	19 (15.3)	55	0 (0)
34. Cantor et al., 2024	USA	NR	Q2M	NR	118	NR	118	0 (0)
35. Prather et al., 2024	USA	1 (minimum)	NR	NR	113	29 (25.7)	113	1 (0.9)
36. Bhayani et al., 2024	USA	2.7 (median)	Q1M and Q2M	NR	100	19 (19.0)	56	0 (0)
Studies with 50–99 virally suppressed participants at baseline (*n* = 24)								
37. Dannenberg et al., 2024	Germany	30 (median)	Q2M	VL > 200 c/mL	96	18 (18.8)	96	0 (0)
38. Pérez et al., 2023	USA	6	Q1M and Q2M	NR	94	39/96 (40.6)^f^	79	1 (1.3)
39. Torralba et al., 2023	Spain	7	Q2M	VL > 50 c/mL	88	17 (19.3)	20	1 (5)
40. Nunnari et al., 2024	Italy	12	Q2M	VL > 50 c/mL	85	11/51 (21.6)^f^	51	1 (2)
41. Antonucci et al., 2024	Italy	13	Q2M	NR	79	24 (30.4)	73	2 (2.7)
42. Nasser et al., 2023	USA	12	Q1M and Q2M	VL > 200 c/mL	80	18/83 (21.7)^f^	72	0 (0)
43. Adachi et al., 2024	Japan	7	NR	VL > 50 c/mL + VL ≥ 200 c/mL	78	2 (2.6)	78	0 (0)
44. Liegeon et al., 2024	USA	8 (median)	Q1M and Q2M	NR	78	36/119 (30.3)^f^	78	1 (1.3)
45. Mesa et al., 2023	USA	2–6 (range)	Q1M and Q2M	NR	76	33/79 (41.8)^f^	63	0 (0)
46. Gandhi et al., 2023 (Ward 86)	USA	8.25 (median)	Q1M and Q2M	NR	76	6 (7.9)	76	0 (0)
47. Shankaran et al., 2024	USA	NR	NR	2 VL > 200 c/mL	75	NR	75	3 (4)
48. Nguyen et al., 2024	USA	9 (median)	Q1M and Q2M	VL > 200 c/mL	73	14 (19.2)	73	3 (4.1)
49. Derrick et al., 2025	USA	6	Q1M and Q2M	VL ≥ 50 c/mL	72	27 (37.5)	53	4 (7.5)
50. Rubenstein et al., 2023	France	15 (median)	Q2M	2 VL ≥ 200 c/mL	72	11 (15.3)	72	1 (1.4)
51. Haser et al., 2024	USA	12	Q1M and Q2M	VL > 200 c/mL	71	12/74 (16.2)^f^	61	1 (1.6)
52. Soffritti et al., 2024	Italy	10.4 (median)	Q2M	2 VL > 20 c/mL or VL > 200 c/mL	68	28/74 (37.8)^f^	68	2 (2.9)
53. Di Biagio and Gaggero, 2024	Italy	6	Q2M	NR	66	NR	66	1 (1.5)
54. Mazzitelli et al., 2023	Italy	3	Q2M	NR	65	17 (26.2)	65	1 (1.5)
55. Yared et al., 2024	USA	12	NR	2 VL ≥ 30 c/mL at least 4 weeks apart	58	10 (17.2)	58	3 (5.2)
56. Tincati et al., 2024	Italy	12	Q2M	NR	57	6 (10.5)	57	0 (0)
57. Iannone et al., 2024	Italy	12	Q2M	NR	53	12/74 (16.2)^f^	53	1 (1.9)
58. Roberts et al., 2023	UK	NR	Q2M	NR	51	1/33 (3.0)^f^	33	0 (0)
59. Ogilvy et al., 2024	USA	2 (minimum)	NR	VL > 200 c/mL	50	7/51 (13.7)^f^	50	0 (0)
60. Vega et al., 2024	USA	2 (minimum)	NR	VL ≥ 200 c/mL	50	8/73 (11.1)^f^	50	0 (0)
Studies with <50 virally suppressed participants at baseline (*n* = 19)								
61. Bowden et al., 2024	Australia	11 (median)	NR	VL ≥ 200 c/mL	42	4/46 (8.7)^f^	42	0 (0)
62. Bobbio et al., 2024	Italy	3	Q2M	NR	42	7 (16.7)	37	0 (0)
63. Schiaroli et al., 2024	Italy	6	Q2M	VL ≥ 20 c/mL	41	5/42 (11.9)^f^	31	0 (0)
64. Rutstein et al., 2024	USA	5 (median)	Q1M and Q2M	VL ≥ 50 c/mL	41	11/47 (23.4)^f^	32	1 (3.1)
65. Koutsoupias et al., 2024	USA	NR	NR	VL > 201 c/mL	39	17/42 (40.5)^f^	39	0 (0)
66. Konishi et al., 2024	Japan	11	Q2M	≥2 VL > 200 c/mL	38	0 (0)	38	0 (0)
67. Carraro et al., 2024	Italy	10.25 (median)	Q2M	NR	36	11 (30.6)	36	0 (0)
68. Rigamonti et al., 2024	Italy	3 (minimum)	Q2M	NR	34	NR	34	0 (0)
69. Kirk et al., 2024	USA	12	Q2M	VL ≥ 200 c/mL	33	9 (27.3)	33	1 (3)
70. Wijesinghe et al., 2023	Canada	NR	NR	VL ≥ 200 c/mL	32	4 (12.5)	32	0 (0)
71. Fernández et al., 2024 (CAR‐GR)	Spain	4	Q2M	VL ≥ 20 c/mL	31	15 (48.4)	31	0 (0)
72. Montalvo et al., 2023	USA	0.25 (minimum)	Q2M	VL ≥ 50 c/mL	30	10 (33.3)	29	0 (0)
73. Williams et al., 2024	USA	33	Q1M and Q2M	VL ≥ 20 c/mL	25	12 (48.0)	25	0 (0)
74. Masich et al., 2023	USA	12	NR	VL > 200 c/mL	24	NR	24	2 (8.3)
75. Ali et al., 2023	UK	9.75 (maximum)	Q2M	NR	18	NR	18	0 (0)
76. Chan et al., 2023	China	5 (median)	Q2M	NR	18	1 (5.6)	18	0 (0)
77. Holland et al., 2023	UK	8	Q2M	NR	15	3 (20.0)	15	0 (0)
78. Spampinato et al., 2023	Italy	2	Q2M	NR	14	1 (7.1)	14	0 (0)
79. Perez et al., 2024	USA	NR	Q2M	VL ≥ 200 c/mL	11	3/15 (20.0)^f^	11	0 (0)

Abbreviations: NR, not reported; Q1M, monthly; Q2M, 2‐monthly; VF, viral failure; VL, viral load.

^a^ The full list of references of included studies is available in Appendix S1.

^b^ We converted durations to months and reported them as standard time points unless otherwise specified in parentheses (when no standard time point was provided).

^c^ We considered female individuals as those reported in observational cohort studies as any of the following: female gender or sex, cis‐gender woman, or if only a male category was provided, we subtracted this from the total number. The percentage was calculated using the number of virally suppressed individuals at baseline as the denominator, unless a different denominator is indicated in the data cell.

^d^ Virally suppressed individuals included in the analysis.

^e^ VF rates were calculated using the total number of virally suppressed individuals at risk of VF as the denominator.

^f^ The denominator differs from the total number of virally suppressed individuals at baseline because gender was not reported discretely for this population (e.g., data provided includes individuals non‐virally suppressed at the switch, or describes the referral or analysis population).

**TABLE 2:** Resistance‐associated mutations in individuals with genotypic information.^a^

RAM data^b^							
	Study	Baseline and/or historic RNA/DNA genotypic information on INI/NNRTI RAMs in those with VF, *n*/*N*; (VF#) *RAMs*	Genotype available at VF, *n*/N	VFs without genotypic evidence of resistance, *n* (*n*/N%, *n*/VF%)	VFs with genotypic evidence of INI RAMs, *n* (*n*/N%, *n*/VF%); (VF#) RAMS	VFs with genotypic evidence of NNRTI RAMs, *n* (*n*/*N*%, *n*/VF%); (VF#) RAMS	VFs with genotypic evidence of dual class resistance (NNRTI and INI RAMs), *n* (*n*/*N*%, *n*/VF%)
1.	Antonucci et al., 2024	0/2	2/2	2 (2.7, 100)	0 (0, 0)	0 (0, 0)	0 (0, 0)
2.	Canavesi et al., 2024	13/14; None None None None 138A None None None None UA None None None K101P, K103S/N, Y181C	13/14	6 (0.8, 46.2)	7 (0.9, 53.8); None None None None **140S,148H** **148R** **G140S, Q148K** **E138E/K, G140S, G163R** None None UA **E138E/K, Q148R** **N155N/H, H51Y** **G140S, Q148H, D232D/N**	6 (0.8, 46.2); None None None None **181I, 190A** **138K, 179I** **K103N, V108I, P225H** None None None UA **E138A, Y188H** **E138K** **V106VI, N348I**	6 (0.8, 46.2)
3.	Deschanvres et al., 2023 (Dat'AIDS Cohort)	0/14^c^	6/14	2 (0.2, 33.3)	3 (0.3, 50); **Q148H/R/K** None **Q148R** **Q148R, R263K**, **N155H** None None	3 (0.3, 50); None **E138K** **E138K** **Y181C**, **E138K** None None	2 (0.2, 33.3)
4.	Gagliardini et al., 2024 (Icona Cohort)	2/2; None None (INI genotype UA)	1/2	0 (0, 0)	1 (0.2, 100); UA **E138A, E157Q**	1 (0.2, 100); UA **K101E**	1 (0.2, 100)
5.	González‐Cordón et al., 2024	2/2; None None	1/2	0 (0, 0)	0 (0, 0); None UA	1 (0.3, 100); **190E** UA	0 (0, 0)
6.	Gutiérrez et al., 2024	2/2^i^ None None	2/2	0 (0, 0)	1 (0.6, 50) **148Q/R**, **263K** None	2 (1.2, 100) **138K** **181C**	1 (0.6, 50)
7.	Haser et al., 2024	0/1	1/1	0 (0, 0)	0 (0, 0)	1 (1.6, 100); **K103N**, **L100I** ^d^	0 (0, 0)
8.	Iannone et al., 2024	0/1	1/1	0 (0, 0)	1 (1.9, 100); **H51Y**	0 (0, 0)	0 (0, 0)
9.	Jongen, 2023 (Dutch ATHENA Cohort)^g^	5/5^h^; 179D None None None None	5/5	0 (0, 0)	4 (0.7, 80); None **138K, 148R** **140S, 148R** **155H** **138K, 148K**	5 (0.9, 100); **101E, 103R**, 179D, **181C** **101E, 138K** **101E** **101E, 138K, 230 L** **90I, 106A, 138K**	4 (0.7, 80)
10.	Jonsson‐Oldenbüttel et al., 2024 (CARLOS)	4/5; UA None None None None	5/5	2 (0.6, 40)	2 (0.6, 40); **Q148R** None **T97A, E138K, Q148R, N155H** None None	3 (0.9, 60); **E138K** None **Y181C** None **K101E**	2 (0.6, 40)
11.	Kirk et al., 2024	1/1; K103N	1/1	0 (0, 0)	1 (3, 100); **E138A**, **G140S**, **Q148H**, **N155H**	1 (3, 100); **L100I**, K103N	1 (3, 100)
12.	Lagi et al., 2024 (LAHIV)	1/2; (unspecified NNRTI RAMs) UA	2/2	1 (0.8, 50)	0 (0, 0)	1 (0.8, 50); 98G, 106I, 108I, 181V None	0 (0, 0)
13.	Liegeon et al., 2024	1/1; None	1/1	0 (0, 0)	1 (1.3, 100); **E138A, G140S, Q148S**	1 (1.3, 100); **K101E, N348I**	1 (1.3, 100)
14.	Maguire et al., 2024	3/3; K103E/Q, V179I, M50I None None	2/3	0 (0, 0)	2 (0.5, 100); UA **Q148R** **N155H**, **R263K**	2 (0.5, 100); UA **Y188L** **K101E**	2 (0.5, 100)
15.	Martín et al., 2024 (RELATIVITY)	5/6; None UA None Q148K/R, E157Q, G140S, L74M/I/F, T97A K103N None	6/6^f^	3 (0.2, 50)	3 (0.2, 50); None **E138K, Q148R, L74LM** None **L100I** None **Y143YS, Q148R**	2 (0.2, 33.3); None **K103N, Y188L** None **K103N** None None	2 (0.2, 33.3)
16.	Masich et al., 2023	1/2; None UA	2/2^f^	2 (8.3, 100)	0 (0, 0)	0 (0, 0)	0 (0, 0)
17.	Mazzitelli et al., 2023	0/1	1/1	1 (1.5, 100)	0 (0, 0)	0 (0, 0)	0 (0, 0)
18.	Muccini et al., 2024 (SCohoLART)	1/4; None UA UA UA	4/4	1 (1.9, 25)	3 (0.6, 75); None **E138E/K, Q148R** **E138K, Q148R** **E157Q**	3 (0.6, 75); None **E138A** **K101P/Q, E138A** **K101E, E138A**	3 (0.6, 75)
19.	Nguyen et al., 2024	3/3; K103N, E138Q None None	3/3	0 (0, 0)	1 (1.4, 33.3); None None **E138E/K, Q148Q/K**	3 (4.1, 100); K103N, E138Q **K103K/R, E138G/R** **M230M/L**	1 (1.4, 33.3)
20.	Pérez et al., 2023	0/1	1/1	0 (0, 0)	0 (0, 0)	1 (1.3, 100); **M230L, V179E**	0 (0, 0)
21.	Pozniak et al., 2024 (COMBINE‐2)	2/3; UA None None	3/3	2 (0.5, 66.7)	0 (0,0)	1 (0.3, 33.3); **E138A** None None	0 (0, 0)
22.	Ring et al., 2024 (SHARE LAI‐net)	0/3^e^	3/3	2 (0.5, 66.7)	0 (0, 0)	1 (0.2, 33.3); None None **K101E**	0 (0, 0)
23.	Rubenstein et al., 2023	0/1^c^	1/1	1 (1.4, 100)	0 (0, 0)	0 (0, 0)	0 (0, 0)
24.	Seang et al., 2023	1/1 None	1/1	0 (0, 0)	1 (0.7, 100) **138K, 148H, 140S, 97A, 74M**	1 (0.7, 100) **101E, 188L, 179I**	1 (0.7, 100)
25.	Serris et al., 2024	4/5 None None T97A None UA	4/5	3 (2.4, 75)	1 (0.8, 20) UA None T97A None UA	0 (0, 0) None UA None UA UA	0 (0, 0)
26.	Shankaran et al., 2024	2/3; None K103N UA	3/3	0 (0, 0)	3 (4, 100); **G140S, L74L/M, T97T/A, Q148H, E138K** **L74I, T97T/A, S147S/G, N155H** **G140G/S, Q148Q/R**	1 (1.3, 33.3); **K101P** None None	1 (1.3, 33.3)
27.	Soffritti et al., 2024	1/2; UA None	2/2	0 (0, 0)	2 (2.9, 100); **G140S**, **Q148H** **Q148R**	2 (2.9, 100); **181I**, **190A** **138K**	2 (2.9, 100)
28.	Yared et al., 2024	1/3; UA UA G140R	3/3	0 (0, 0)	3 (5.2, 100); **E92K, Q146R** **Q148K, E138K** **Q148R**	3 (5.2, 100); **E138K**, **M230I** **Y188L, V106I** **E138K**	3 (5.2, 100)

Abbreviations: DNA, deoxyribonucleic acid; INI, integrase inhibitor, NR, not reported; NNRTI, non‐nucleoside reverse transcriptase inhibitor; RAMs, resistance‐associated mutations; RNA, ribonucleic acid; UA, unavailable; VF, viral failure.

^a^ The full list of references of included studies is available in Appendix S1.

^b^ RAMs not listed under INI or NNRTI in the Stanford Algorithm were excluded. Bolded RAMs were not present in baseline/historical genotypes prior to VF. n/N RAMs rate was calculated using the total number of virally suppressed individuals at risk of VF as the denominator.

^c^ Absence of NNRTI RAMs at baseline may be inferred due to country‐specific eligibility requirements for LA‐I CAB+RPV.

^d^ These RAMs were not detected before VF.

^e^ None had RPV DRMs at baseline.

^f^ Baseline or historic RAMs were excluded from genotype results at VF.

^g^ Data were also extracted from: van Welzen BJ, et al. Virological failure after switch to long‐acting cabotegravir and rilpivirine injectable therapy: An in‐depth analysis. Clin Infect Dis. 2024;79 (1):189–95.

^h^ Baseline resistance testing only available for reverse transcriptase RAMs.

^i^ Historical genotype only available for NNRTI RAMs.

**TABLE 3:** Post‐VF regimens and re‐suppression rates.^a^

Post‐VF data				
	Study	Post‐VF regimen described (*n*/N)	Post‐VF regimen^b^ Drug class; (VF#) regimen	Re‐suppression^c^ *n*/*N*, (%); (VF#) Y/N/UA
1.	Antonucci et al., 2024	2/2	INI; BIC/TAF/FTC BIC/TAF/FTC	1/1 (100) Y UA
2.	Dawiec et al., 2024	2/2	PI, INI; FTC/TAF/DRV/c FTC/TDF+DTG	2/2 (100)
3.	Deschanvres et al., 2023 (Dat'AIDS)	6/14^d^	LA‐I CAB+RPV	5/6 (83.3)^d^
4.	Eron et al., 2024 (Trio Health)	2/2	INI, PI; DRV/c/F/TAF BIC/FTC/TAF	1/2 (50) N Y
5.	Fernández‐Hinojal et al., 2023	3/3	LA‐I CAB+RPV	3/3 (100)
6.	Gagliardini et al., 2024 (Icona)	2/2	LA CAB+RPV, INI; LA‐I CAB+RPV FTC/TAF/BIC	1/2 (50) Y N
7.	González‐Cordón et al., 2024	2/2	2 LA‐I CAB+RPV	2/2 (100)
8.	Haser et al., 2024	1/1	LA‐I CAB+RPV	1/1 (100)
9.	Hsu et al., 2024 (OPERA)	25/25	10 INI; 10 LA‐I CAB+RPV 4 Multi‐tablet 1 Therapeutic gap	15/19^d^ (78.9) 6 VFs without FU
10.	Iannone et al., 2024	1/1	PI; TAF/FTC/DRV/c	NR
11.	Jongen, 2023 (ATHENA)^e^	5/5	3 PI, 1 LA‐I CAB+RPV, 1 INI; TAF/FTC/DRV/c INI‐based triple therapy+MVC TAF/FTC/DRV/c TAF/FTC/DRV/c LA CAB+RPV	4/4 (100) Y Y Y Y UA
12.	Jonsson‐Oldenbüttel et al., 2024 (CARLOS)	5/5	3 PI, 2 INI; DRV/COBI/FTC/TAF BIC/FTC/TAF DRV/COBI/FTC/TAF BIC/FTC/TAF DRV/COBI/FTC/TAF	NR
13.	Kirk et al., 2024	1/1	PI; DRV/c/FTR/DOR	1/1 (100)
14.	Lagi et al., 2024 (LAHIV)	2/2	PI, INI; T/F/DRVc 3TC/DTG	1/1 (100) Y UA
15.	Liegeon et al., 2024	1/1	PI; TAF/FTC/DRV/c	1/1 (100)
16.	Maguire et al., 2024	3/3	2 PI, 1 INI; BIC/TAF/FTC DRV/c/TAF/FTC DRV/c/TAF/FTC	3/3 (100)
17.	Martín et al., 2024 (RELATIVITY)	6/6	4 PI, 2 INI; DTG/3TC DRVc/FTC/TAF BIC/FTC/TAF DRVc/FTC/TAF DRVc/FTC/TAF DRVc/FTC/TAF	5/6 (83.3) Y N Y Y Y Y
18.	Masich et al., 2023	2/2	INI, LA‐I CAB+RPV BIC/TAF/FTC LA‐I CAB+RPV	1/1 (100) UA Y
19.	Muccini et al., 2024 (SCohoLART)	4/4	3 PI, 1 INI; BIC/F/TAF DRV/c/F/ TAF DRV/c/F/ TAF DRV/c/F/ TAF	4/4 (100)
20.	Pozniak et al., 2024 (COMBINE‐2)	3/3	PI; DRV/c/FTC/TAF ABC/3TC/DRV/r DRV/c/FTC/TAF	2/2 (100) UA Y Y
21.	Ring et al., 2024 (SHARE LAI‐net)	3/3	PI, INI, NNRTI; BIC/F/TAF DOR/3TC/TDF DRV/c/FTC/TAF	3/3 (100)
22.	Seang et al., 2023	1/1	PI; TDF/FTC+DRV/r	0/1 (0)
23.	Serris et al., 2024	5/5	4 INI, PI TDF/FTC+DRV/r ABC/3TC/DTG TAF/FTC/BIC TAF/FTC/BIC TAF/FTC/BIC	5/5 (100)
24.	Shankaran et al., 2024	3/3	PI; (specific regimens NR)	3/3 (100)
25.	Soffritti et al., 2024	2/2	PI, Multi‐tablet; DTG/RPV/MVC DRV/c/FTC/TAF	1/1 (100) Y UA

Abbreviations: FU, follow‐up; INI, integrase inhibitor; LA‐I CAB+RPV, long‐acting cabotegravir and rilpivirine; N, no; NR, not reported; NNRTI, non‐nucleoside reverse transcriptase inhibitor; PI, protease inhibitor; UA, unavailable; VF, viral failure; Y, yes.

^a^ The full list of references of included studies is available in Appendix S1.

^b^ When multiple post‐VF regimens were listed, we reported the one provided at the moment of re‐suppression (if data available), or otherwise the first regimen listed.

^c^ Based on definition of suppression or individuals reported as ‘re‐suppressed’ by individual studies.

^d^ Data not matched to specific VFs.

^e^ Data were also extracted from: van Welzen BJ, et al. Virological failure after switch to long‐acting cabotegravir and rilpivirine injectable therapy: An in‐depth analysis. Clin Infect Dis. 2024;79 (1):189–95.

Seventy of 79 cohorts (89%) reported demographic information on sex or gender, and all but one included individuals described as ‘female’ or ‘women’. In mixed cohorts, which included both virally suppressed and non‐suppressed individuals, baseline demographic data were not always discretely reported, so the total proportion of virally suppressed ‘females’ included could not be determined. Study‐level reporting on gender is described in Table 1. Data on inclusion of transgender or gender non‐conforming individuals were seldom provided and the categories of ‘sex’ and ‘gender’ were often conflated or not defined. Additional characteristics of study populations such as country, pre‐existing historic RAMS or DNA evidence of baseline resistance (where available) are included (Tables 1 and 2).

### VF definitions

VF definitions differed among OCS, and 27 of the 79 cohorts did not specify a definition. The most common VF definition was an unconfirmed VL greater than a specified VL threshold (e.g., 50 or 200 c/mL); 13.9% (11/79) of definitions were based on confirmatory VLs and 17.7% (14/79) used other definitions (e.g., unconfirmed viraemia followed by discontinuation or ART modification) (Table 4).

**TABLE 4:** Summary table of study durations, key virological definitions, and virological outcomes: Virological failure, resistance‐associated mutations, post‐VF regimens and suppression rates.

OCS follow‐up duration, *n* (*n*/*N*%)	
Through 6 months	30 (38.0%)
Less than 6 months	17 (21.5%)
Through 12 months	12 (15.2%)
Over 12 months	10 (12.7%)
Not reported	10 (12.7%)
VF definitions used, *n* (*n*/*N*%)	
Unconfirmed VL	27 (34.2%)
Not reported	27 (34.2%)
Other	14 (17.7%)
Confirmatory VL	11 (13.9%)
OCS that describe presence/absence of VF, *n*	79
OCS with VF events	48
OCS without VF events	31
Individuals at risk of VF,^a^ *n*	13 899
VF events, *n*	172
OCS reporting genotypic data on presence/absence of RAMs at VF, *n*	28
Individuals at risk of RAMs,^a^ *n*	7987
VF events with genotypic data on presence/absence of RAMs, *n*	80
VF events with RAMs identified	52
VF events where no RAMs were observed	28
RAMS by drug class,^b^ *n*	
NNRTI RAMs observed	45
INI RAMs observed	40
NNRTI and INI RAMs observed	33
Number of RAMs at VF per individual	
1 NNRTI RAMs	21
2 NNRTI RAMs	18
3 NNRTI RAMs	4
4+ NNRTI RAMs	2
1 INI RAMs	12
2 INI RAMs	18
3 INI RAMs	5
4+ INI RAMs	5
OCS with post‐VF regimen data, *n*	25
Individuals at risk of VF,^a^ *n*	8521
VF events with post‐VF regimen data provided, *n*	92
Regimen type	
Protease‐inhibitor, *n* (*n*/*N*%)	31 (33.7%)
INI‐based	29 (31.5%)
LA‐I CAB+RPV	25 (27.2%)
Multi‐tablet	5 (5.4%)
NNRTI‐based	1 (1.1%)
Therapeutic gap	1 (1.1%)
OCS reporting on re‐suppression outcomes post‐VF, *n*	23
Individuals at risk of VF,^a^ *n*	8117
VF events with known re‐suppression outcome, *N*	74
Re‐suppressed, *n* (*n*/*N*%)	65 (87.8%)
Not re‐suppressed	9 (12.2%)

Abbreviations: INI, major integrase inhibitor; LA‐I CAB+RPV, long‐acting injectable cabotegravir and rilpivirine; NNRTI, non‐nucleoside reverse transcriptase inhibitor; OCS, observational cohort studies; RAMs, resistance‐associated mutations; VF, virological failure; VL, viral load.

^a^ Individuals included in the VF analysis.

^b^ Categories are not mutually exclusive.

### Duration of follow‐up

Thirty of 79 (38.0%) OCS reported time on LA‐I CAB+RPV through 6 months, 21.5% (17/79) < 6 months, 15.2% (12/79) through 12 months, 12.7% (10/79) > 12 months and 12.7% (10/79) did not report duration (Table 4). Durations were variably reported by investigators as means, medians or time points in relation to number of injections received.

### Risk of bias

Seven studies (9%) were identified as inadequate quality (scoring less than 50%, and showing high risk of bias), and the remaining 72 studies (91%) were identified as moderate quality or above (scoring more than 50% and showing low risk of bias). The main source of bias was a lack of adjustment for different lengths of follow‐up of patients (34/79 studies), not reporting all important adverse events (49/79 studies) and a lack of accurate outcome measures, for example not including a definition of VF (52/79 studies) (Table S8).

### VF events

Out of the 79 OCS comprising 13 899 individuals at risk of VF, 172 individuals experienced VF events across 48 OCS (Table 4).

### RAMs at VF

Genotypic information at VF was provided in 28 OCS, which included 7987 individuals at risk of VF (Table 4). Out of the 80 VF events with genotypic information available at the time of the event, NNRTI mutations were identified in 45 cases, INIs in 40 cases, and dual‐class resistance in 33 cases. Notably, 28 VF events did not exhibit any resistance; 46.7% (21/45) of individuals with NNRTI RAMs had a single NNRTI mutation, 40.0% (18/45) had two mutations and 13.3% (6/45) had three or more mutations. Twelve of 40 (30.0%) of individuals with INI RAMs had one INI mutation, 40.0% (18/40) had two mutations and 25.0% (10/40) had three or more mutations. Table 2 provides specific details on RAMs per patient and per study.

According to the Stanford HIV Drug Resistance Database, the most prevalent NNRTI‐associated mutations were E138A/K (K101E/P/Q, K103K/R/N and Y181C/I), and the most prevalent INI‐associated mutations were Q148H/Q/K/R/S, E138A/E/K, G140G/S, L74L/M/I, T97T/A and R263K. Interpretation of HIV DNA sequences bearing E138K in reverse transcriptase and E138K, G140S and R263K in integrase requires caution, as they may arise through the terminally mutagenic effects of APOBEC (apolipoprotein B mRNA editing catalytic polypeptide‐like)‐3G/F rather than represent the effects of drug selection. Concerning genotypic susceptibility to future INI‐containing regimens, substitutions at integrase positions 148 and 263 are considered to have the most significant impact on susceptibility to bictegravir and dolutegravir.

### Historic or DNA evidence of baseline resistance in individuals with VF

Data pertaining to historical resistance or the presence /absence of INI and/or NNRTI RAMs in baseline genotypes were provided for 55 VF events across 20 studies (Table 2). Of these 55 individuals, 43 exhibited no evidence of resistance, 9 exhibited NNRTI resistance, and 4 exhibited INI resistance. The most prevalent baseline NNRTI mutation was K103 mixtures, detected in six individuals. Notably, there was one individual with unspecified NNRTI mutations.

### Management of VF

Twenty‐five studies involving 8521 individuals described 92 post‐VF treatment regimens (Table 4). The most prevalent regimens prescribed were PI‐based (31, 33.7%), followed by oral INI‐based (29, 31.5%); 27% of individuals (*n* = 25) continued LA‐I CAB+RPV post‐VF, while multi‐tablet regimens were prescribed in 5.4% (five individuals). Re‐suppression outcomes were reported in 23 studies, involving 74 events among 8117 individuals. Among the 74 VF events, post‐VF re‐suppression occurred in 87.8% (65/74).

## DISCUSSION

A recent manuscript by Jongen et al. presented findings from the ATHENA cohort, a longstanding observational nationwide HIV cohort in the Netherlands. In this study, 525 individuals who had commenced LA‐I CAB+RPV while virally suppressed were matched 1:2 with 1170 individuals who continued oral antiretroviral therapy. The authors observed that in these virally suppressed individuals, the risk of virological control loss was no different in those who received LA‐I CAB+RPV than those who received oral therapy [30].

In this systematic literature review spanning 79 OCS and 13 countries, including data from 13 899 virally suppressed individuals who transitioned to LA‐I CAB+RPV, we confirm that VF is very uncommon in clinical practice. At the time of VF, INI resistance, the most significant consequence of emerging resistance, occurs but is not invariable.

Building upon previous reports, we also provide a detailed summary of the commonest NNRTI, INI and dual class resistance patterns. The selection of an appropriate regimen in the presence of INI resistance has become increasingly pertinent for the field. Recent reports on emergent resistance at VF on dolutegravir‐based ART, which is used algorithmically as first‐ and second‐line therapy in WHO regions, have prompted discussions on the sequencing of subsequent therapies [31]. Consequently, our findings that clinicians utilize both PI‐based regimens and INI‐based regimens post‐VF are relevant to this regimen and the broader field. Another noteworthy finding is that clinicians continued LA‐I CAB+RPV post‐VF in more than a quarter of VF cases. Based on interviews conducted during implementation studies, this may possibly indicate preference from individuals on LA‐I CAB+RPV to remain on injectables [11].

Importantly, re‐suppression (where reported) was high, although the follow‐up period post‐suppression was heterogeneous and inconsistently reported.

Our summary of baseline evidence of mutations (i.e., historical RNA or proviral DNA resistance testing) that predates the switch to LA‐I CAB+RPV shows that this was rarely performed and implies that clinicians are relying on the evaluation of clinical history. Among the 55 individuals in whom it was reported, most (43/55) did not have baseline or historical resistance, and the presence of INI resistance was very low (4/55). The prevalence of NNRTI resistance was slightly higher (9/55). It is interesting to note that K103 mixtures, which do not affect RPV susceptibility, were the commonest of baseline mutations described.

These data, which reflects clinical practice, are reassuring and should alleviate concerns among clinicians who are clearly using clinical history to inform their practice without the need to perform expensive proviral DNA testing which is often unavailable and does not necessarily reflect resistance. These data support LA‐I CAB+RPV use in virally suppressed individuals seeking a novel treatment strategy, both for convenience and/or to address the more apparent stigma‐related concerns associated with oral therapy.

One limitation of our study was the inability to utilize the GRADE tool to evaluate the certainty of evidence, as it is specifically designed for outcomes that are measured by effect sizes. The heterogeneity of VF definition and variable follow‐up time do not warrant presenting a VF rate from these OCS. However, the substantial denominator and the exclusive focus on the virally suppressed population may enhance confidence in the regimen, particularly considering that VF was infrequently reported, as observed in clinical trials and implementation science studies, ranging between 0% and 2.41% [4, 5, 6, 7, 8, 9, 10, 11].

The quality of evidence presented is variable, with both length of time and cohort size varying significantly between studies. Out of 79 OCS, only 10% included more than 500 participants and only 4% included more than 1000 and 38% of OCS included less than 100 participants (see Table 1). As we progress and more evidence from clinical practice emerges, we are hopeful that follow‐up time and cohort size in published OCS will improve the evidence quality.

The sole focus on the virally suppressed population is a strength and limitation in that our results should only be interpreted in that context. The Hill group meta‐analysis, which included a few studies in non‐suppressed individuals, confirms that the risk of developing VF in individuals with detectable viraemia may be higher [25]. Post‐VF suppression rates in viraemic populations may also vary, although this has not been reported. Notably, most OCS did not describe known risk factors such as BMI, A6 subtype and RPV RAMs, which precludes assessment of those at highest risk of VF in clinical practice [12]. Almost all OCS included in this review are based in high‐income countries where LA‐I CAB+RPV is available and supported within the clinic infrastructure. While the CARES trial and the IMPAACT study describe reassuring outcomes in the context of a public health approach in African countries, the lack of availability in these settings precludes reporting observational data and therefore cannot be extrapolated beyond high‐resource settings [7, 32]. The heterogeneity of duration, VF definitions, and inconsistencies in reporting on mutations and post‐VF regimens reduce the number of individuals studied.

The authors recommend that all future published reports on OCS describe VF using a common VF definition, and report key clinical variables including emergent resistance, post‐VF regimens and re‐suppression outcomes. Longer‐term observational cohort data, data in non‐virally suppressed individuals and evidence in resource‐constrained settings remain important evidence gaps.

## AUTHOR CONTRIBUTIONS

C.O. M.S. and K.R. conceptualized the study and acquired the study funding. The methodology was determined by C.O., A.E., K.R. and M.S. Elizabeth Ward and Claire Snowball from Inizio Medical performed searches according to the protocol. Ying Jean, Jamie Shorrock and Christopher Dee from Inizio Medical performed the primary screening steps. A.E. and C.O. were responsible for secondary study screening and extraction. A.E., C.O. and M.D. were responsible for quality assessment. A.E., M.D. and C.O. were responsible for data analysis. Study findings were interpreted by C.O. and K.R. Visualization of the data was conducted by A.E., M.D. and C.O. The original draft of the manuscript was written by C.O., A.E., K.R. and M.D. Reviews and edits of subsequent drafts were conducted by all co‐authors.

## FUNDING INFORMATION

This study was funded by a grant from ViiV Healthcare.

## CONFLICT OF INTEREST STATEMENT

Chloe Orkin has received honoraria for advisory boards, lectureships, and travel sponsorships from Janssen, Gilead Sciences, ViiV Healthcare, MSD and Bavarian Nordic and has received research grants from Janssen, Gilead Sciences, ViiV Healthcare, MSD and AstraZeneca. Kyle Ring has received speaker and consultation fees from ViiV Healthcare and is an investigator on drug trials sponsored by Gilead Sciences and MSD. Melanie Smuk, Alexa Elias and Megan Devonald have no interests to declare.

## Supporting information


**Data S1.** Supporting information.

## Data Availability

Data will be made available from the corresponding author on reasonable request.
