# Supplementary material for: Long‐acting injectable cabotegravir and rilpivirine in observational cohort studies: A systematic review on virological failure, resistance and re‐suppression outcomes in virally suppressed individuals living with HIV
Source: HIV Med. 2025 Jun 13;26(8):1267–88. doi: 10.1111/hiv.70057 (PMC12315068; doi:10.1111/hiv.70057)
Supplement: Supplementary file 1 — Data S1. Supporting information. [file HIV-26-1267-s001.docx]

**Appendix**

**This appendix is supplement to:**

Long-acting injectable cabotegravir and rilpivirine in observational cohort studies: a systematic review on virological failure, resistance, and re-suppression outcomes in virally suppressed individuals living with HIV.

Supplement Version Date: 10/3/2025

Contents

[Supplemental Methods 3](#_Toc198038064)

[Search Strategy 3](#_Toc198038065)

[Table S1. Search Strategy used via Embase 3](#_Toc198038066)

[Table S2. Search Strategy used via *Pubmed* 4](#_Toc198038067)

[Table S3. Search Strategy used via *Cochrane* 5](#_Toc198038068)

[Table S4. List of 23 HIV-related congresses from which abstracts, posters, and oral presentations were hand-searched 5](#_Toc198038069)

[Inclusion criteria 6](#_Toc198038070)

[Table S5. PICOS Framework 6](#_Toc198038071)

[Data Extraction 6](#_Toc198038072)

[Table S6. Data extraction fields 6](#_Toc198038073)

[Quality Assessment 7](#_Toc198038074)

[Table S7. Modified Downs and Black Checklist 7](#_Toc198038075)

[Supplemental Results 9](#_Toc198038076)

[Table S8. Risk of bias assessment using modified Downs and Black checklist 9](#_Toc198038077)

[Table S9. Study characteristics and data on VF, resistance, post-VF regimens and re-suppression in virally suppressed individuals initiating LA-I CAB+RPV in observational cohort studies 14](#_Toc198038078)

[Full reference list of observational cohort studies included in the systematic review 23](#_Toc198038079)

[Appendix References 31](#_Toc198038080)

# Supplemental Methods

## Search Strategy

### Table S1. Search Strategy used via Embase

| **No.** | **Query** | **Subject** |
| --- | --- | --- |
| 1 | 'Human immunodeficiency virus'/exp OR 'human immunodeficiency virus infection'/exp | **Disease** |
| 2 | 'human immunodeficiency virus' OR 'human immunedeficiency virus' OR (human AND immun* AND (virus* OR infection*)) |  |
| 3 | 'hiv' OR 'hiv1' OR 'hiv2' OR 'hivi' OR 'hivii' |  |
| 4 | 'acquired immune deficiency syndrome'/exp |  |
| 5 | 'aids' OR 'acquired immunodeficiency syndrome' OR 'acquired immunedeficiency syndrome' OR (acquired AND immun* AND (virus* OR infection* OR syndrome*)) |  |
| 6 | #1 OR #2 OR #3 OR #4 OR #5 |  |
| 7 | 'cabotegravir plus rilpivirine'/exp OR (cabotegravir NEAR/3 rilpivirine) OR (cab NEAR/2 (rpv OR ril)) OR cabenuva OR vocabria OR rekambys | **Intervention** |
| 8 | #6 AND #7 | **Combination** |
|  |  |  |
|  |  |  |
| 9 | #8 NOT (('animal'/exp NOT 'human'/exp) OR ‘In Vitro Study’/exp OR ‘Cell Culture Technique’/exp OR ‘Cell Line’/exp OR ‘ex vivo’:ab,ti OR ‘in vitro’:ab,ti OR ‘ cell line’:ab,ti OR ‘ cell culture’:ab,ti OR ‘animal cell’:ab,ti OR ‘animal tissue’:ab,ti OR ‘animal experiment’:ab,ti OR ‘animal health’:ab,ti OR ‘animal model’:ab,ti OR ‘bird’:ab,ti OR ‘canine’:ab,ti OR ‘dog’:ab,ti OR ‘goat’:ab,ti OR ‘mouse’:ab,ti OR ‘mammal’:ab,ti OR ‘mice’:ab,ti OR ‘murine’:ab,ti OR ‘non human’:ab,ti OR ‘nonhuman’:ab,ti OR ‘rat’:ab,ti OR ‘veterinary’:ab,ti) | **Limited to humans** |
| 10 | #9 NOT ('review':de OR 'editorial':de OR 'addresses':de OR 'biography':de OR 'comment':de OR 'directory':de OR 'festschrift':de OR 'interview':de OR 'legislation':de OR 'news':de OR 'newspaper article':de OR 'patient education handout':de OR 'consensus development conference':de OR 'consensus development conference, nih':de OR 'practice guideline':de OR 'Controlled Clinical Trial':de OR ATLAS:ti OR FLAIR:ti OR 'Phase 1*':ti OR 'Phase I*':ti OR 'Phase 2*':ti OR 'Phase II*':ti OR 'Phase 3*':ti OR 'Phase III*':ti) | **Removal of irrelevant publication types** |
| 11 | #10 AND ('article'/it OR 'article in press'/it OR 'conference abstract'/it OR 'conference paper'/it OR 'conference review'/it OR 'letter'/it) | **Restriction to manuscripts or congress abstracts** |
| 12 | #11 NOT [medline]/lim | **Restriction to Embase-only records** |

### Table S2. Search Strategy used via *Pubmed*

| **No.** | **Query** | **Subject** |
| --- | --- | --- |
| 1 | "HIV"[Mesh] OR "HIV Infections"[Mesh] | **Disease** |
| 2 | "hiv" OR "hiv1" OR "hiv2" OR "hivi" OR "hivii" |  |
| 3 | "human immunodeficiency virus" OR "human immunedeficiency virus" OR ((“human” AND “immun*”) AND ("virus*" OR “infection*”)) |  |
| 4 | "Acquired Immunodeficiency Syndrome"[Mesh] |  |
| 5 | "AIDS" OR "acquired immunodeficiency syndrome" OR "acquired immunedeficiency syndrome" OR ("acquired" AND "immun*" AND ("virus*" OR "infection*" OR "syndrome*")) |  |
| 6 | #1 OR #2 OR #3 OR #4 OR #5 |  |
| 7 | "cabotegravir, rilpivirine drug combination" [Supplementary Concept] OR "rilpivirine cabotegravir"[tiab:~3] OR "CAB RPV"[tiab:~2] OR "CAB RIL"[tiab:~2] OR Cabenuva OR Vocabria OR "Rekambys" | **Intervention** |
| 8 | #6 AND #7 | **Combination** |
| 9 | #8 NOT (("Animals"[MeSH] NOT "Humans"[MeSH]) OR "In Vitro Techniques"[Mesh] OR "Cell Culture Techniques"[Mesh] OR "Cell Line"[Mesh] OR "ex vivo"[tiab] OR "in vitro"[tiab] OR "cell line"[tiab] OR "cell culture"[tiab] OR "animal cell"[tiab] OR "animal tissue"[tiab] OR "animal experiment"[tiab] OR "animal health"[tiab] OR "animal model"[tiab] OR "bird"[tiab] OR "canine"[tiab] OR "dog"[tiab] OR "goat"[tiab] OR "mouse"[tiab] OR "mammal"[tiab] OR "mice"[tiab] OR "murine"[tiab] OR "non human"[tiab] OR "nonhuman"[tiab] OR "rat"[tiab] OR "veterinary"[tiab]) | **Limited to humans** |
|  |  |  |
| 10 |  | **Removal of irrelevant publication types** |
|  | #9 NOT ("Review"[Publication Type] OR "Editorial"[Publication Type] OR "biography"[Publication Type] OR "comment"[Publication Type] OR "directory"[Publication Type] OR "festschrift"[Publication Type] OR "interview"[Publication Type] OR "legislation"[Publication Type] OR "news"[Publication Type] OR "newspaper article"[Publication Type] OR "patient education handout"[Publication Type] OR "consensus development conference"[Publication Type] OR "consensus development conference, nih"[Publication Type] OR "practice guideline"[Publication Type] OR "Controlled Clinical Trial"[Publication Type] OR "ATLAS"[ti] OR "FLAIR"[ti] OR "Phase 1*"[ti] OR "Phase I*"[ti] OR "Phase 2*"[ti] OR "Phase II*"[ti] OR "Phase 3*"[ti] OR "Phase III*"[ti]) |  |
|  |  |  |
|  |  |  |

### Table S3. Search Strategy used via *Cochrane*

| **No.** | **Query** | **Subject** |
| --- | --- | --- |
| 1 | MeSH descriptor: [HIV] explode all trees | **Disease** |
| 2 | MeSH descriptor: [HIV Infections] explode all trees |  |
| 3 | hiv or "hiv-1" or "hiv 1" or "hiv-2" or "hiv 2" or hiv1 or hiv2 or "hiv-i" or "hiv-ii" or "hiv infection" or "hiv-infection" or ("hiv NEXT infect*") or (hiv and infect*) |  |
| 4 | MeSH descriptor: [Acquired Immunodeficiency Syndrome] explode all trees |  |
| 5 | aids or "acquired immunodeficiency syndrome" or "acquired immunedeficiency syndrome" or ("acquired and immun*" and (virus* or infection* OR syndrome*)) |  |
| 6 | #1 or #2 or #3 or #4 or #5 |  |
| 7 | (cabotegravir NEAR/3 rilpivirine) OR (CAB NEAR/2 RPV) OR (CAB NEAR/2 RIL) OR 'Cabenuva' OR Vocabria OR Rekambys | **Intervention** |
| 8 | (#6 AND #7) | **Combination** |

### Table S4. List of 23 HIV-related congresses from which abstracts, posters, and oral presentations were hand-searched

| 1 | Afravih |
| --- | --- |
| 2 | Asian Conference on Hepatitis and AIDS (ACHA) |
| 3 | Australasian HIV & AIDS Conference (ASHM) |
| 4 | National Conference of AIDS Society of India (ASICON) |
| 5 | British Association for Sexual Health and HIV (BASHH) |
| 6 | British HIV Association (BHIVA) |
| 7 | Canadian Conference on HIV/AIDS Research (CAHR) |
| 8 | Conference on Retroviruses and Opportunistic Infections (CROI) |
| 9 | European AIDS Clinical Society (EACS) |
| 10 | European Meeting on HIV and Hepatitis |
| 11 | Grupo de Estudio del SIDA-SEIMC (GeSIDA) |
| 12 | HIV Glasgow |
| 13 | HIV & Hepatitis in the Americas (HIV/HEP) |
| 14 | IDWeek |
| 15 | International AIDS Society/International AIDS Conference (IAS/IAC) |
| 16 | Italian Conference on AIDS and Antiviral Research (ICAR) |
| 17 | International Conference on AIDS and STIs in Africa (ICASA) |
| 18 | International Congress on Infectious Diseases (ICID) |
| 19 | International Workshop on HIV Drug Resistance and Treatment Strategies |
| 20 | The Professional Society for Health Economics and Outcomes Research (ISPOR) |
| 21 | Japanese Society for AIDS Research (JSAR) |
| 22 | Kenya Association of Physicians (KAP) |
| 23 | Société Française De Lutte Contre Le Sida (SFLS) |

## Inclusion criteria

Inclusion and exclusion criteria for Phase 1 of the OUTCOMES Study were developed based on the following items, organised using the PICOS (Population, Intervention, Comparator, Outcome, Study design) Framework.

### Table S5. PICOS Framework

| Population: | Adults and adolescents living with HIV-1 who are virally suppressed |
| --- | --- |
| Intervention: | Long-acting injectable cabotegravir and rilpivirine |
| Comparator: | Any or none |
| Outcomes: | Virological outcomes at follow-up (e.g., reporting of presence or absence of virological failure, viral suppression (or non-suppression), or other viral load endpoint) |
| Study Design: | Observational cohort studies (e.g., prospective or retrospective observational cohort studies, case reports, analysis of hospital records) including > 10 virally suppressed people at switch. |

## Data Extraction

Information on study characteristics, VF, RAMs, and post-VF regimen in the virally suppressed population was extracted and stored in Microsoft Excel based on the following criteria:

### Table S6. Data extraction fields

| Study Characteristics | VF | RAM data | Post-VF data |
| --- | --- | --- | --- |
| - Study name or first author and year - Study countries - Follow-up time (months) - Dose - VF definition - Number of virally suppressed people at baseline - Number of virally suppressed people at risk of VF | - Number of VF events, *n (%)* | - Genotype available for baseline and/or historic INI/NNRTI RAMs for those with VF,   *n/N;*  *VF#) RAMs*   - Genotype available at VF,   *n/N*   - VFs with available genotypes with No RAMs,   *n (n/N%, n/VF%)*   - VFs with available genotypes with INI RAMs,   *n (n/N%, n/VF%);*  *VF#) RAMS*   - VFs with available genotypes with NNRTI RAMs,   *n (n/N%, n/VF%);*  *VF#) RAMS*   - VFs with available genotypes with NNRTI & INI RAMs,   *n (n/N%, n/VF%)* | - Post-VF regimen available *(n/N)* - Post-VF regimen,   *drug class;*  *VF#) regimen*   - Re-suppression *n/N, (%);*   *VF#) Y/N/UA* |

## Quality Assessment

Out of 27 items present in the original Downs and Black Checklist,^1^ 16 were excluded in our modified version. We modified the tool in line with previous reviews which also appraised observational cohort studies and studies without an experimental study design.^2,3^ Excluded items concerned randomisation and blinding, measures of effect and statistical tests, and study arms, as this review focused on outcomes observed through descriptive statistics in real-world patients electing to receive LA-I CAB+RPV. Each question received a score of 0 or 1. Similarly to former reviews using the modified checklist,^2,4^ studies fulfilling at least 50% of items were considered moderate or above scientific quality. The assessment was completed by A.E. and cross-checked by M.D. Disagreements in scoring were resolved by a third author (C.O.).

### Table S7. Modified Downs and Black Checklist

| **Section I: Reporting** *(Scoring per question: Yes=1, No=0)* |
| --- |
| 1. Is the hypothesis/ aim/ objective of the study clearly described?  2. Are the main outcomes to be measured clearly described in the Introduction or Methods section?  3. Are the characteristics of the patients included in the study clearly described ?  4. Are the interventions of interest clearly described?  5. Are the main findings of the study clearly described?  6. Have all important adverse events that may be a consequence of the intervention been reported?  7. Have the characteristics of patients lost to follow-up been described? |
| **Section II: External Validity** *(Scoring per question: Yes=1, No=0, Unable to determine=0)* |
| 8. Were the staff, places, and facilities where the patients were treated, representative of the treatment the majority of patients receive? |
| **Section III: Internal Validity- Bias** *(Scoring per question: Yes=1, No=0, Unable to determine=0)* |
| 9. In trials and cohort studies, do the analyses adjust for different lengths of follow-up of patients, or in case-control studies, is the time period between the intervention and outcome the same for cases and controls ?  10. Were the main outcome measures used accurate (valid and reliable)? |
| **Section IV: Internal validity- confounding (selection bias)** *(Scoring per question: Yes=1, No=0, Unable to determine=0)* |
| 11. Were losses of patients to follow-up taken into account? |

| Supplemental ResultsTable S8. Risk of bias assessment using modified Downs and Black checklist | | | | | | | | | | | | | | | |
| --- | --- | --- | --- | --- | --- | --- | --- | --- | --- | --- | --- | --- | --- | --- | --- |
|  | Study | 1. Is the hypothesis/ aim/ objective of the study clearly described? | 2. Are the main outcomes to be measured clearly described in the Introduction or Methods section? | 3. Are the characteristics of the patients included in the study clearly described? | 4. Are the interventions of interest clearly described? | | 5. Are the main findings of the study clearly described? | 6. Have all important adverse events that may be a consequence of the intervention been reported? | 7. Have the characteristics of patients lost to follow-up been described? | 8. Were the staff, places, and facilities where the patients were treated, representative of the treatment the majority of patients receive? | 9. Do the analyses adjust for different lengths of follow-up of patients? | 10. Were the main outcome measures used accurate (valid and reliable)? | 11. Were losses of patients to follow-up taken into account? | **Total, n (%)** | **Grade** |
| **1** | Adachi et al., 2024 | 1 | 1 | 1 | 0 | | 1 | 0 | 1 | 1 | 1 | 1 | 1 | **9 (82)** | Moderate or above |
| **2** | Ali et al., 2023 | 1 | 0 | 1 | 1 | | 0 | 0 | 0 | 1 | 0 | 0 | 1 | **5 (45)** | Inadequate |
| **3** | An Chiu et al., 2024 | 1 | 1 | 1 | 0 | | 0 | 1 | 1 | 1 | 1 | 1 | 1 | **9 (82)** | Moderate or above |
| **4** | Antonucci et al., 2024 | 1 | 1 | 0 | 1 | | 1 | 1 | 1 | 1 | 1 | 0 | 1 | **9 (82)** | Moderate or above |
| **5** | Bhayani et al., 2024 | 1 | 1 | 0 | 1 | | 0 | 1 | 1 | 1 | 0 | 0 | 1 | **7 (64)** | Moderate or above |
| **6** | Bobbio et al., 2024 | 1 | 1 | 1 | 1 | | 1 | 1 | 1 | 1 | 1 | 0 | 1 | **10 (91)** | Moderate or above |
| **7** | Bowden et al., 2024 | 1 | 1 | 1 | 0 | | 1 | 1 | 1 | 1 | 0 | 1 | 1 | **9 (82)** | Moderate or above |
| **8** | Canavesi et al., 2024 | 1 | 1 | 0 | 1 | | 1 | 1 | 1 | 1 | 0 | 0 | 1 | **8 (73)** | Moderate or above |
| **9** | Cantor et al., 2024 | 1 | 0 | 1 | 1 | | 1 | 0 | 0 | 1 | 0 | 0 | 0 | **5 (45)** | Inadequate |
| **10** | Carraro et al., 2024 | 0 | 1 | 0 | 1 | | 1 | 1 | 0 | 1 | 0 | 0 | 0 | **5 (45)** | Inadequate |
| **11** | Chan et al., 2023 | 1 | 1 | 1 | 1 | | 0 | 0 | 1 | 1 | 0 | 0 | 1 | **7 (64)** | Moderate or above |
| **12** | Dannenberg et al., 2024 | 1 | 1 | 1 | 1 | | 1 | 1 | 1 | 1 | 0 | 1 | 1 | **10 (91)** | Moderate or above |
| **13** | Dawiec et al., 2024 | 0 | 1 | 1 | 1 | | 0 | 1 | 1 | 1 | 1 | 0 | 1 | **8 (73)** | Moderate or above |
| **14** | Derrick et al., 2024 | 1 | 1 | 1 | 1 | | 1 | 0 | 1 | 1 | 1 | 1 | 1 | **10 (91)** | Moderate or above |
| **15** | Deschanvres et al., 2023 (Dat’AIDSCohort) | 1 | 1 | 1 | 1 | | 1 | 1 | 1 | 1 | 0 | 1 | 1 | **10 (91)** | Moderate or above |
| **16** | Di Biagio & Gaggero, 2024 | 1 | 1 | 0 | 1 | | 1 | 1 | 1 | 1 | 1 | 0 | 0 | **8 (73)** | Moderate or above |
| **17** | Eron et al., 2024 (Trio) | 1 | 1 | 1 | 1 | | 1 | 0 | 0 | 1 | 0 | 1 | 1 | **8 (73)** | Moderate or above |
| **18** | Fernández et al., 2024 (CAR-GR) | 1 | 1 | 1 | 1 | | 1 | 1 | 1 | 1 | 1 | 1 | 1 | **11 (100)** | Moderate or above |
| **19** | Fernández-Hinojal et al., 2023 | 1 | 1 | 1 | 1 | | 1 | 0 | 1 | 1 | 0 | 1 | 1 | **9 (82)** | Moderate or above |
| **20** | Ferrara et al., 2024 (LARES Cohort) | 1 | 1 | 0 | 1 | | 1 | 0 | 0 | 1 | 1 | 1 | 1 | **8 (73)** | Moderate or above |
| **21** | Fessler et al., 2024 | 0 | 1 | 0 | 1 | | 1 | 0 | 0 | 1 | 0 | 1 | 1 | **6 (55)** | Moderate or above |
| **22** | Gagliardini et al., 2024 (IconaCohort) | 1 | 1 | 1 | 1 | | 1 | 1 | 1 | 1 | 0 | 1 | 1 | **10 (91)** | Moderate or above |
| **23** | Gandhi et al. 2023 | 1 | 1 | 1 | 1 | | 1 | 1 | 0 | 1 | 0 | 0 | 1 | **8 (73)** | Moderate or above |
| **24** | González-Cordón et al., 2024 | 1 | 1 | 0 | 1 | | 1 | 0 | 0 | 1 | 1 | 1 | 1 | **8 (73)** | Moderate or above |
| **25** | Gutiérrez et al., 2024 | 1 | 1 | 1 | 1 | | 1 | 0 | 0 | 1 | 0 | 1 | 1 | **8 (73)** | Moderate or above |
| **26** | Haser et al., 2024 | 1 | 1 | 0 | 1 | | 1 | 1 | 1 | 1 | 1 | 1 | 1 | **10 (91)** | Moderate or above |
| **27** | Hessamfar et al., 2024 (ANRS CO3 AquiVIH NA Cohort) | 1 | 1 | 1 | 1 | | 1 | 1 | 1 | 1 | 1 | 1 | 1 | **11 (100)** | Moderate or above |
| **28** | Hidalgo-Tenorio et al., 2024 (CABO-CHANCE) | 1 | 1 | 1 | 1 | | 0 | 1 | 0 | 1 | 1 | 0 | 1 | **8 (73)** | Moderate or above |
| **29** | Hill et al., 2025 | 1 | 1 | 1 | 1 | | 1 | 0 | 0 | 1 | 0 | 1 | 1 | **8 (73)** | Moderate or above |
| **30** | Holland et al., 2023 | 1 | 0 | 1 | 1 | | 0 | 0 | 1 | 1 | 1 | 0 | 1 | **7 (64)** | Moderate or above |
| **31** | Hsu et al., 2024 (OPERA) | 1 | 1 | 1 | 1 | | 1 | 0 | 0 | 1 | 0 | 1 | 1 | **8 (73)** | Moderate or above |
| **32** | Iannone et al., 2024 | 1 | 1 | 0 | 1 | | 1 | 0 | 1 | 1 | 1 | 0 | 1 | **8 (73)** | Moderate or above |
| **33** | Jonnsson-Oldenbuttel et al., 2024 (CARLOS) | 1 | 1 | 1 | 1 | | 1 | 1 | 1 | 1 | 1 | 1 | 1 | **11 (100)** | Moderate or above |
| **34** | Jongen et al., 2025 (Dutch ATHENACohort)^$^ | 1 | 1 | 1 | 1 | | 1 | 0 | 0 | 1 | 0 | 1 | 1 | **8 (73)** | Moderate or above |
| **35** | Kirk et al., 2024 | 1 | 1 | 1 | 1 | | 1 | 1 | 1 | 1 | 1 | 1 | 1 | **11 (100)** | Moderate or above |
| **36** | Konishi et al., 2024 | 1 | 1 | 1 | 1 | | 1 | 1 | 1 | 1 | 1 | 1 | 1 | **11 (100)** | Moderate or above |
| **37** | Koutsoupias et al., 2024 | 0 | 1 | 0 | 0 | | 1 | 0 | 1 | 1 | 0 | 1 | 1 | **6 (55)** | Moderate or above |
| **38** | Lagi et al., 2024 (LAHIV) | 1 | 1 | 1 | 1 | | 0 | 1 | 1 | 1 | 0 | 1 | 1 | **9 (82)** | Moderate or above |
| **39** | Liegeon et al., 2024 | 1 | 1 | 1 | 1 | | 0 | 1 | 1 | 1 | 0 | 0 | 1 | **8 (73)** | Moderate or above |
| **40** | Liu et al., 2024 | 1 | 1 | 1 | 0 | | 1 | 0 | 1 | 1 | 0 | 1 | 1 | **8(73)** | Moderate or above |
| **41** | Maguire et al., 2024 | 1 | 1 | 1 | 1 | | 1 | 0 | 0 | 1 | 0 | 1 | 0 | **7 (64)** | Moderate or above |
| **42** | Martin et al., 2024 (RELATIVITY) | 1 | 1 | 1 | 1 | | 1 | 1 | 1 | 1 | 1 | 0 | 1 | **10 (91)** | Moderate or above |
| **43** | Masich et al., 2023 | 0 | 1 | 1 | 0 | | 1 | 1 | 1 | 1 | 1 | 1 | 1 | **9 (82)** | Moderate or above |
| **44** | Matone et al., 2024 | 1 | 1 | 1 | 1 | | 1 | 1 | 1 | 1 | 0 | 1 | 1 | **10 (91)** | Moderate or above |
| **45** | Mazzitelli et al., 2023 | 1 | 1 | 1 | 1 | | 1 | 1 | 1 | 1 | 1 | 0 | 1 | **10 (91)** | Moderate or above |
| **46** | Mesa et al. 2023 | 1 | 1 | 1 | 1 | | 1 | 0 | 1 | 1 | 0 | 0 | 1 | **8 (73)** | Moderate or above |
| **47** | Montalvo et al., 2023 | 1 | 1 | 1 | 1 | | 1 | 1 | 1 | 1 | 0 | 1 | 1 | **10 (91)** | Moderate or above |
| **48** | Muccini et al., 2024 (SCohoLART) | 1 | 1 | 1 | 1 | | 1 | 1 | 1 | 1 | 0 | 1 | 1 | **10 (91)** | Moderate or above |
| **49** | Nasser et al., 2023 | 1 | 1 | 1 | 1 | | 1 | 1 | 1 | 1 | 1 | 1 | 1 | **11 (100)** | Moderate or above |
| **50** | Nguyen et al., 2024 | 1 | 1 | 1 | 1 | | 1 | 1 | 1 | 1 | 0 | 1 | 1 | **10 (91)** | Moderate or above |
| **51** | Nunnari et al., 2024 | 1 | 1 | 1 | 1 | | 1 | 0 | 0 | 1 | 1 | 1 | 1 | **9 (82)** | Moderate or above |
| **52** | Ogilvy et al., 2024 | 1 | 1 | 1 | 0 | | 1 | 0 | 0 | 1 | 0 | 1 | 1 | **7 (64)** | Moderate or above |
| **53** | Palacios et al., 2024 (CARIPLA) | 1 | 1 | 1 | 1 | | 0 | 1 | 1 | 1 | 0 | 1 | 1 | **9 (82)** | Moderate or above |
| **54** | [Perez et al., 2024](https://academic.oup.com/ofid/article/11/11/ofae640/7833466) | 1 | 1 | 1 | 1 | | 1 | 1 | 1 | 1 | 0 | 1 | 1 | **10 (91)** | Moderate or above |
| **55** | Pérez S, et al., 2023 | 1 | 1 | 1 | 1 | | 0 | 1 | 1 | 1 | 1 | 0 | 1 | **9 (82)** | Moderate or above |
| **56** | Pozniak et al., 2024 (COMBINE-2) | 1 | 1 | 1 | 1 | | 1 | 0 | 0 | 1 | 0 | 1 | 1 | **8 (73)** | Moderate or above |
| **57** | Prather et al., 2024 | 1 | 1 | 1 | 0 | | 0 | 0 | 1 | 1 | 0 | 0 | 1 | **6 (55)** | Moderate or above |
| **58** | Psomas et al., 2023 | 1 | 1 | 1 | 1 | | 1 | 1 | 1 | 1 | 1 | 0 | 1 | **10 (91)** | Moderate or above |
| **59** | Ramirez et al., 2023 | 1 | 0 | 1 | 1 | | 1 | 1 | 1 | 1 | 0 | 1 | 1 | **9 (82)** | Moderate or above |
| **60** | Ribera et al., 2023 | 1 | 1 | 1 | 1 | | 0 | 1 | 1 | 1 | 1 | 0 | 1 | **9 (82)** | Moderate or above |
| **61** | Rigamonti et al., 2024 | 1 | 1 | 0 | 1 | | 0 | 1 | 1 | 1 | 0 | 0 | 1 | **7 (64)** | Moderate or above |
| **62** | Ring et al., 2024 (SHARE LAI-net) | 1 | 1 | 1 | 1 | | 1 | 1 | 1 | 1 | 0 | 1 | 1 | **10 (91)** | Moderate or above |
| **63** | Roberts et al., 2023 | 1 | 0 | 0 | 1 | | 1 | 0 | 0 | 1 | 0 | 0 | 1 | **5 (45)** | Inadequate |
| **64** | Rubenstein et al. 2023 | 1 | 1 | 1 | 1 | | 1 | 1 | 1 | 1 | 0 | 1 | 1 | **10 (91)** | Moderate or above |
| **65** | Rutstein et al., 2025 | 1 | 1 | 1 | 1 | | 1 | 1 | 1 | 1 | 0 | 1 | 1 | **10 (91)** | Moderate or above |
| **66** | Schiaroli et al., 2024 | 1 | 1 | 0 | 1 | | 1 | 1 | 0 | 1 | 1 | 1 | 1 | **9 (82)** | Moderate or above |
| **67** | Schneider et al., 2024 (BEYOND) | 1 | 1 | 1 | 1 | | 1 | 1 | 1 | 1 | 1 | 1 | 1 | **11 (100)** | Moderate or above |
| **68** | Seang et al., 2023 | 1 | 1 | 1 | 1 | | 1 | 1 | 1 | 1 | 1 | 1 | 1 | **11 (100)** | Moderate or above |
| **69** | Serris et al., 2024 | 1 | 1 | 1 | 1 | | 1 | 1 | 1 | 1 | 0 | 1 | 1 | **10 (91)** | Moderate or above |
| **70** | Shankaran et al., 2024 | 1 | 1 | 0 | 0 | | 1 | 0 | 0 | 1 | 0 | 1 | 0 | **5 (45)** | Inadequate |
| **71** | Soffritti et al., 2024 | 1 | 1 | 1 | 1 | | 1 | 1 | 1 | 1 | 0 | 1 | 1 | **10 (91)** | Moderate or above |
| **72** | Spampinato et al. 2023 | 1 | 1 | 0 | 1 | | 0 | 0 | 1 | 1 | 1 | 0 | 1 | **7 (64)** | Moderate or above |
| **73** | Taramasso et al., 2024 (SCOLTA) | 1 | 1 | 1 | 1 | | 0 | 1 | 1 | 1 | 0 | 0 | 1 | **8 (73)** | Moderate or above |
| **74** | Tincati et al., 2024 | 1 | 0 | 1 | 1 | | 0 | 0 | 0 | 1 | 1 | 0 | 0 | **5 (45)** | Inadequate |
| **75** | Torralba et al., 2023 | 1 | 1 | 1 | 1 | | 1 | 1 | 1 | 1 | 1 | 1 | 1 | **11 (100)** | Moderate or above |
| **76** | Vega et al., 2024 | 1 | 1 | 1 | 0 | | 1 | 0 | 1 | 1 | 0 | 1 | 1 | **8 (73)** | Moderate or above |
| **77** | Wijesinghe et al., 2023 | 1 | 1 | 0 | 0 | | 1 | 0 | 0 | 1 | 0 | 1 | 0 | **5 (45)** | Inadequate |
| **78** | Williams et al., 2024 | 0 | 1 | 1 | 1 | | 1 | 1 | 1 | 1 | 1 | 1 | 1 | **10 (91)** | Moderate or above |
| **79** | Yared et al., 2024 | 1 | 1 | 1 | 0 | | 1 | 1 | 1 | 1 | 1 | 1 | 1 | **10 (91)** | Moderate or above |
| **Total** | | 73/79 | 73/79 | 62/79 | | 67/79 | 62/79 | 49/79 | 57/79 | 79/79 | 34/79 | 52/79 | 72/79 |  |  |

### Table S9. Study characteristics and data on VF, resistance, post-VF regimens and re-suppression in virally suppressed individuals initiating LA-I CAB+RPV in observational cohort studies

| **Study characteristics** | | | | | | | | **VF** | RAM data^&^ | | | | | | **Post-VF data** | | |
| --- | --- | --- | --- | --- | --- | --- | --- | --- | --- | --- | --- | --- | --- | --- | --- | --- | --- |
| Study | Study country/ countries | Follow-up time, months^@^ | Dose | VF definition | Virally suppressed at baseline, N | Female, n (n/N%)* | Virally suppressed at risk of VF ^‡^ | VF, n (%)^☥^ | Baseline and /or historic RNA/DNA genotypic information on INI/NNRTI RAMs in those with VF,  *n/N;*  *VF#) RAMs* | Genotype available at VF, *n/N* | VFs without genotypic evidence of resistance, *n (n/N%, n/VF%)* | VFs with genotypic evidence of INI RAMs,  *n (n/N%, n/VF%);*  *VF#) RAMS* | VFs with genotypic evidence of NNRTI RAMs,  *n (n/N%, n/VF%);*  *VF#) RAMS* | VFs with genotypic evidence of dual class resistance (NNRTI & INI RAMs),  *n (n/N%, n/VF%)* | Post-VF regimen described (n/N) | Post-VF regimen^©^  *Drug class;*  *VF#) regimen* | Resuppression^ⵜ^ *n/N, (%);*  *VF#) Y/N/UA* |
| 1. Hsu et al., 2024 (OPERA) | USA | NR | Q1M & Q2M | 2 VL≥200 c/mL or VL≥200 c/mL + discontinuation | 1362 | 237 (17.4) | 1,293 | 25 (1.9) |  |  |  |  |  |  | 25/25 | 10 INI;  10 LA-I CAB+RPV  4 Multi-tablet  1 Therapeutic gap | 15/19^✖^ (78.9)  VFs without FU |
| 1. Martín et al., 2024 (RELATIVITY) | Spain | 13 | Q2M | NR | 1285 | 183 (14.3) | 1265 | 6 (0.5) | 5/6;  1) None  2) UA  3) None  4) Q148K/R, E157Q, G140S, L74M/I/F, T97A  5) K103N  6) None | 6/6^#^ | 3 (0.2, 50) | 3 (0.2, 50);  1) None  2) **E138K, Q148R, L74LM**  3) None  4) **L100I**  5) None  6) **Y143YS, Q148R** | 2 (0.2, 33.3);  1) None  2) **K103N, Y188L**  3) None  4) **K103N**  5) None  6) None | 2 (0.2, 33.3) | 6/6 | 4 PI, 2 INI;  1) DTG/3TC  2) DRVc/FTC/TAF  3) BIC/FTC/TAF  4) DRVc/FTC/TAF  5) DRVc/FTC/TAF  6) DRVc/FTC/TAF | 5/6 (83.3)  1) Y  2) N  3) Y  4) Y  5) Y  6) Y |
| 1. Deschanvres et al., 2023 (Dat’AIDS) | France | 6.3 (median) | Q2M | 2 VL>50 c/mL or  VL>200 c/mL | 1134 | 237 (20.9) | 1134 | 14 (1.2) | 0/14**^^^** | 6/14 | 2 (0.2, 33.3) | 3 (0.3, 50);  1) **Q148H/R/K**  2) None  3) **Q148R**  4) **Q148R, R263K**, **N155H**  5) None  6) None | 3 (0.3, 50);  1) None  2) **E138K**  3) **E138K**  4) **Y181C**, **E138K**  5) None  6) None | 2 (0.2, 33.3) | 6/14^✖^ | LA-I CAB+RPV | 5/6 (83.3)^✖^ |
| 1. Canavesi et al., 2024 | Italy | 5.23 (mean) | Q2M | NR | 758 | NR | 758 | 14 (1.8) | 13/14;  1) None  2) None  3) None  4) None  5) 138A  6) None  7) None  8) None  9) None  10) UA  11) None  12) None  13) None  14) K101P, K103S/N, Y181C | 13/14 | 6 (0.8, 46.2) | 7 (0.9, 53.8);  1) None  2) None  3) None  4) None  5) **140S,148H**  6) **148R**  7) **G140S, Q148K**  8) **E138E/K, G140S, G163R**  9) None  10) None  11) UA  12) **E138E/K, Q148R**  13) **N155N/H, H51Y**  14) **G140S, Q148H, D232D/N** | 6 (0.8, 46.2);  1) None  2) None  3) None  4) None  5) **181I, 190A**  6) **138K, 179I**  7) **K103N, V108I, P225H**  8) None  9) None  10) None  11) UA  12) **E138A, Y188H**  13) **E138K**  14) **V106VI, N348I** | 6 (0.8, 46.2) |  |  |  |
| 1. Jongen, 2023 (Dutch ATHENA Cohort) | Netherlands | 9.6 (median) | Q2M | VL>200 c/mL | 619 | 61 (9.9) | 588 | 5 (0.9) | 5/5**^£^**;  1) 179D  2) None  3) None  4) None  5) None | 5/5 | 0 (0, 0) | 4 (0.7, 80);  1) None  2) **138K, 148R**  3) **140S, 148R**  4) **155H**  5) **138K, 148K** | 5 (0.9, 100);  1) **101E, 103R,** 179D, **181C**  2) **101E, 138K**  3) **101E**  4) **101E, 138K, 230L**  5) **90I, 106A, 138K** | 4 (0.7, 80) | 5/5 | 3 PI, 1 LA-I CAB+RPV, 1 INI;  1) TAF/FTC/DRV/c  2) INI-based triple therapy+MVC  3) TAF/FTC/DRV/c  4) TAF/FTC/DRV/c  5) LA CAB+RPV | 4/4 (100)  1) Y  2) Y  3) Y  4) Y  5) UA |
| 1. Ring et al., 2024 (SHARE LAI-net) | UK | 7.5 (median) | Q2M | 2 VL≥200 c/mL | 518 | 150 (28.9) | 433 | 3 (0.7) | 0/3^@@^ | 3/3 | 2 (0.5, 66.7) | 0 (0, 0) | 1 (0.2, 33.3);  1) None  2) None  3) **K101E** | 0 (0, 0) | 3/3 | PI, INI, NNRTI;  1) BIC/F/TAF  2) DOR/3TC/TDF  3) DRV/c/FTC/TAF | 3/3 (100) |
| 1. Muccini et al.,, 2024 (SCohoLART) | Italy | 13.1 (median) | Q2M | 2 VL≥50 c/mL or VL≥1000 c/mL | 514 | 47 (9.1) | 514 | 4 (0.8) | 1/4;  1) None  2) UA  3) UA  4) UA | 4/4 | 1 (1.9, 25) | 3 (0.6, 75);  1) None  2) **E138E/K, Q148R**  3) **E138K, Q148R**  4) **E157Q** | 3 (0.6, 75);  1) None  2) **E138A**  3) **K101P/Q, E138A**  4) **K101E, E138A** | 3 (0.6, 75) | 4/4 | 3 PI, 1 INI;  1) BIC/F/TAF  2) DRV/c/F/ TAF  3) DRV/c/F/ TAF  4) DRV/c/F/ TAF | 4/4 (100) |
| 1. Gagliardini et al., 2024 (Icona) | Italy | NR | Q2M | 2 VL>50 c/mL or VL>1000 c/mL + ART change | 506 | 57 (11.3) | 506 | 2 (0.4) | 2/2;  1) None  2) None (INI genotype UA) | 1/2 | 0 (0, 0) | 1 (0.2, 100);  1) UA  2) **E138A, E157Q** | 1 (0.2, 100);  1) UA  2) **K101E** | 1 (0.2, 100) | 2/2 | LA CAB+RPV, INI;  1) LA-I CAB+RPV  2) FTC/TAF/BIC | 1/2 (50)  1) Y  2)N |
| 1. Pozniak et al., 2024 (COMBINE-2) | Switzerland, Germany, France, Spain, Netherlands | 3 (median) | Q2M | 2 VL≥200 c/mL or VL≥200c/mL + discontinuation | 472 | 53 (11.2) | 374 | 3 (0.8) | 2/3;  1) UA  2) None  3) None | 3/3 | 2 (0.5, 66.7) | 0 (0,0) | 1 (0.3, 33.3);  1) **E138A**  2) None  3) None | 0 (0, 0) | 3/3 | PI;  1) DRV/c/FTC/TAF  2) ABC/3TC/DRV/r  3) DRV/c/FTC/TAF | 2/2 (100)  1) UA  2) Y  3) Y |
| 1. Maguire et al., 2024 | USA | 8.2 (median) | Q1M & Q2M | 2 VL≥200 c/mL | 374 | 94 (25.1) | 374 | 3 (0.8) | 3/3;  1) K103E/Q, V179I, M50I  2) None  3) None | 2/3 | 0 (0, 0) | 2 (0.5, 100);  1) UA  2)**Q148R**  3) **N155H**, **R263K** | 2 (0.5, 100);  1) UA  2) **Y188L**  3) **K101E** | 2 (0.5, 100) | 3/3 | 2 PI, 1 INI;  1)BIC/TAF/FTC  2)DRV/c/TAF/FTC  3)DRV/c/TAF/FTC | 3/3 (100) |
| 1. Taramasso et al., 2024 (SCOLTA) | Italy | 10 (median) | Q2M | NR | 370 | 90/377 (23.9)^¥^ | 370 | 4 (1.1) |  |  |  |  |  |  |  |  |  |
| 1. Hessamfar et al., 2024 (ANRS CO3 AquiVIH NA Cohort) | France | 12 | Q2M | VL>1000 c/mL or 2 VL>50 c/mL | 362 | 98/374 (26.2) ^¥^ | 362 | 9 (2.5) |  |  |  |  |  |  |  |  |  |
| 1. Jonsson-Oldenbüttel et al., 2024 (CARLOS) | Germany | 12 | Q2M | 2 VL≥200 c/mL or VL≥200 c/mL + discontinuation | 351 | 19 (5.4) | 351 | 5 (1.4) | 4/5;  1) UA  2) None  3) None  4) None  5) None | 5/5 | 2 (0.6, 40) | 2 (0.6, 40);  1) **Q148R**  2) None  3) **T97A, E138K, Q148R, N155H**  4) None  5) None | 3 (0.9, 60);  1) **E138K**  2) None  3) **Y181C**  4) None  5) **K101E** | 2 (0.6, 40) | 5/5 | 3 PI, 2 INI;  1)DRV/COBI/FTC/TAF  2) BIC/FTC/TAF  3)DRV/COBI/FTC/TAF  4) BIC/FTC/TAF  5)DRV/COBI/FTC/TAF | NR |
| 1. González-Cordón et al., 2024 | Spain | 7 | Q2M | 2 VL>50 cp/mL | 313 | 25/318 (7.9)^¥^ | 313 | 2 (0.6) | 2/2;  1) None  2) None | 1/2 | 0 (0, 0) | 0 (0, 0);  1) None  2) UA | 1 (0.3, 100);  1) **190E**  2) UA | 0 (0, 0) | 2/2 | 2 LA-I CAB+RPV | 2/2 (100) |
| 1. Palacios et al., 2024 (CARIPLA) | Spain | 7.8 (median) | Q2M | VL ≥200 c/ml | 281 | 36 (12.8) | 264 | 7 (2.7) |  |  |  |  |  |  |  |  |  |
| 1. Eron et al., 2024 (Trio Health) | USA | 10 (median) | Q1M & Q2M | 2 VL≥200 c/mL or 1 VL≥200 c/mL+ discontinuation within 4 months of last injection | 278 | 47/268 (17.5) ^¥^ | 221 | 2 (0.9) |  |  |  |  |  |  | 2/2 | INI, PI;  1) DRV/c/F/TAF  2) BIC/FTC/TAF | 1/2 (50)  1) N  2) Y |
| 1. Seang et al., 2023 | France | 12 | Q2M | 2 VL> 200 c/mL | 270 | 44/283 (15.5) ^¥^ | 137 | 1 (0.7) | 1/1  None | 1/1 | 0 (0, 0) | 1 (0.7, 100)  **138K, 148H, 140S, 97A, 74M** | 1 (0.7, 100)  **101E, 188L, 179I** | 1 (0.7, 100) | 1/1 | PI;  TDF/FTC+DRV/r | 0/1 (0) |
| 1. Ramirez, 2023 (Swiss HIV Cohort) | Switzerland | 14 (median) | Q2M | 2 VL>50 c/mL | 264 | 48 (18.2) | 264 | 1 (0.4) |  |  |  |  |  |  |  |  |  |
| 1. Hill et al., 2025 | USA | 14.5 (median) | Q1M & Q2M | 2 VL> 200 c/mL | 252 | 43/287 (15.0) ^¥^ | 252 | 2 (0.8) |  |  |  |  |  |  |  |  |  |
| 1. Liu et al., 2024 | USA | NR | NR | VL>200 c/mL | 233 | 132 (56.7) | 53 | 1 (1.9) |  |  |  |  |  |  |  |  |  |
| 1. Schneider et al., 2024 (BEYOND) | USA | 12 | Q1M & Q2M | 2 VL≥200 c/mL or VL≥200 c/mL + discontinuation within 3 months | 233 | 28 (12) | 206 | 2 (1.0) |  |  |  |  |  |  |  |  |  |
| 1. Hidalgo-Tenorio et al., 2024 (CABO-CHANCE) | Spain | 7 | Q2M | NR | 224 | 22 (9.8) | 149 | 0 (0) |  |  |  |  |  |  |  |  |  |
| 1. Gutiérrez et al., 2024 | Spain | 11.1 (median) | Q2M | 2 VL≥200 c/mL or VL ≥1000 c/mL | 173 | 25 (14.5) | 173 | 2 (1.2) | 2/2^∞^  1) None  2) None | 2/2 | 0 (0, 0) | 1 (0.6, 50)  1) **148Q/R**, **263K**  2) None | 2 (1.2, 100)  1) **138K**  2) **181C** | 1 (0.6, 50) |  |  |  |
| 1. Ferrara, 2024 (LARES) | Italy | 11 | Q2M | VL>50 c/mL | 172 | 27/176 (15.3) ^¥^ | 42 | 0 (0) |  |  |  |  |  |  |  |  |  |
| 1. Dawiec et al., 2024 | Poland | 24 | Q2M | NR | 170 | NR | 170 | 2 (1.2) |  |  |  |  |  |  | 2/2 | PI, INI;  1) FTC/TAF/DRV/c  2) FTC/TDF+DTG | 2/2 (100) |
| 1. An Chiu et al., 2024 | USA | 6 | NR | VL>50 c/mL | 169 | 29 (17.2) | 128 | 5 (3.9) |  |  |  |  |  |  |  |  |  |
| 1. Fessler et al., 2024 | USA | NR | Q1M & Q2M | VL ≥200 c/mL | 153 | NR | 141 | 6 (4.3) |  |  |  |  |  |  |  |  |  |
| 1. Psomas et al., 2023 | France | 18 | Q2M | NR | 142 | 31 (21.8) | 142 | 1 (0.7) |  |  |  |  |  |  |  |  |  |
| 1. Matone et al., 2024 | Italy | 10.75 (median) | Q2M | 2 VL> 50 c/mL 4 weeks  apart or VL> 1000 c/mL | 138 | 32 (23.2) | 138 | 0 (0) |  |  |  |  |  |  |  |  |  |
| 1. Fernández-Hinojal et al., 2023 | Spain | 4.1 (median) | Q2M | VL ≥50 c/mL | 135 | 17/139 (43.6) ^¥^ | 128 | 3 (2.3) |  |  |  |  |  |  | 3/3 | LA-I CAB+RPV | 3/3 (100) |
| 1. Lagi et al., 2024 (LAHIV) | Italy | 7 (median) | Q2M | 2 VL>50 c/mL or 1 VL>1000 c/mL + ART change | 129 | 23 (17.8) | 129 | 2 (1.6) | 1/2;  1) (unspecified NNRTI RAMs)  2) UA | 2/2 | 1 (0.8, 50) | 0 (0, 0) | 1 (0.8, 50);  1) 98G, 106I, 108I, 181V  2) None | 0 (0, 0) | 2/2 | PI, INI;  1) T/F/DRVc  2) 3TC/DTG | 1/1 (100)  1) Y  2) UA |
| 1. Serris et al., 2024 | France | 10.5 (median) | Q2M | VL>200 c/mL+ discontinuation, or 2 VL>50 c/mL | 126 | 33 (26.2) | 126 | 5 (4) | 4/5  1) None  2) None  3) T97A  4) None  5) UA | 4/5 | 3 (2.4, 75) | 1 (0.8, 20)  1) UA  2) None  3) T97A  4) None  5)UA | 0 (0, 0)  1) None  2) UA  3) None  4) UA  5)UA | 0 (0, 0) | 5/5 | 4 INI, PI  1) TDF/FTC+DRV/r  2) ABC/3TC/DTG  3) TAF/FTC/BIC  4) TAF/FTC/BIC  5) TAF/FTC/BIC | 5/5 (100) |
| 1. Ribera et al., 2023 | Spain | 5 | Q2M | NR | 124 | 19 (15.3) | 55 | 0 (0) |  |  |  |  |  |  |  |  |  |
| 1. Cantor et al., 2024 | USA | NR | Q2M | NR | 118 | NR | 118 | 0 (0) |  |  |  |  |  |  |  |  |  |
| 1. Prather et al., 2024 | USA | 1 (minimum) | NR | NR | 113 | 29 (25.7) | 113 | 1 (0.9) |  |  |  |  |  |  |  |  |  |
| 1. Bhayani et al., 2024 | USA | 2.7 (median) | Q1M & Q2M | NR | 100 | 19 (19.0) | 56 | 0 (0) |  |  |  |  |  |  |  |  |  |
| 1. Dannenberg et al., 2024 | Germany | 30 (median) | Q2M | VL>200 c/ml | 96 | 18 (18.8) | 96 | 0 (0) |  |  |  |  |  |  |  |  |  |
| 1. Pérez et al., 2023 | USA | 6 | Q1M & Q2M | NR | 94 | 39/96 (40.6)^¥^ | 79 | 1 (1.3) | 0/1 | 1/1 | 0 (0, 0) | 0 (0, 0) | 1 (1.3, 100);  **M230L, V179E** | 0 (0, 0) |  |  |  |
| 1. Torralba et al., 2023 | Spain | 7 | Q2M | VL> 50 c/mL | 88 | 17 (19.3) | 20 | 1 (5) |  |  |  |  |  |  |  |  |  |
| 1. Nunnari et al., 2024 | Italy | 12 | Q2M | VL>50 c/mL | 85 | 11/51 (21.6)^¥^ | 51 | 1 (2) |  |  |  |  |  |  |  |  |  |
| 1. Antonucci et al., 2024 | Italy | 13 | Q2M | NR | 79 | 24 (30.4) | 73 | 2 (2.7) | 0/2 | 2/2 | 2 (2.7, 100) | 0 (0, 0) | 0 (0, 0) | 0 (0, 0) | 2/2 | INI;  1) BIC/TAF/FTC  2) BIC/TAF/FTC | 1/1 (100)  1) Y  2) UA |
| 1. Nasser et al., 2023 | USA | 12 | Q1M & Q2M | VL>200 c/mL | 80 | 18/83 (21.7)^¥^ | 72 | 0 (0) |  |  |  |  |  |  |  |  |  |
| 1. Adachi et al., 2024 | Japan | 7 | NR | VL>50 c/mL + VL≥200 c/mL | 78 | 2 (2.6) | 78 | 0 (0) |  |  |  |  |  |  |  |  |  |
| 1. Liegeon et al., 2024 | USA | 8 (median) | Q1M & Q2M | NR | 78 | 36/119 (30.3) ^¥^ | 78 | 1 (1.3) | 1/1;  None | 1/1 | 0 (0, 0) | 1 (1.3, 100);  **E138A, G140S, Q148S** | 1 (1.3, 100);  **K101E, N348I** | 1 (1.3, 100) | 1/1 | PI;  TAF/FTC/DRV/c | 1/1 (100) |
| 1. Mesa et al., 2023 | USA | 2-6  (range) | Q1M & Q2M | NR | 76 | 33/79 (41.8)^¥^ | 63 | 0 (0) |  |  |  |  |  |  |  |  |  |
| 1. Gandhi et al., 2023 | USA | 8.25 (median) | Q1M & Q2M | NR | 76 | 6 (7.9) | 76 | 0 (0) |  |  |  |  |  |  |  |  |  |
| 1. Shankaran et al., 2024 | USA | NR | NR | 2 VL>200 c/mL | 75 | NR | 75 | 3 (4) | 2/3;  1) None  2) K103N  3) UA | 3/3 | 0 (0, 0) | 3 (4, 100);  1) **G140S, L74L/M, T97T/A, Q148H, E138K**  2) **L74I, T97T/A, S147S/G, N155H**  3) **G140G/S, Q148Q/R** | 1 (1.3, 33.3);  1) **K101P**  2) None  3) None | 1 (1.3, 33.3) | 3/3 | PI;  (specific regimens NR) | 3/3 (100) |
| 1. Nguyen et al., 2024 | USA | 9 (median) | Q1M & Q2M | VL>200 c/mL | 73 | 14 (19.2) | 73 | 3 (4.1) | 3/3;  1) K103N, E138Q  2) None  3) None | 3/3 | 0 (0, 0) | 1 (1.4, 33.3);  1) None  2) None  3) **E138E/K, Q148Q/K** | 3 (4.1, 100);  1) K103N, E138Q  2) **K103K/R, E138G/R**  3) **M230M/L** | 1 (1.4, 33.3) |  |  |  |
| 1. Derrick et al., 2025 | USA | 6 | Q1M & Q2M | VL≥50 c/mL | 72 | 27 (37.5) | 53 | 4 (7.5) |  |  |  |  |  |  |  |  |  |
| 1. Rubenstein et al., 2023 | France | 15 (median) | Q2M | 2 VL≥200 c/mL | 72 | 11 (15.3) | 72 | 1 (1.4) | 0/1**^^^** | 1/1 | 1 (1.4, 100) | 0 (0, 0) | 0 (0, 0) | 0 (0, 0) |  |  |  |
| 1. Haser et al., 2024 | USA | 12 | Q1M & Q2M | VL>200 c/mL | 71 | 12/74 (16.2)^¥^ | 61 | 1 (1.6) | 0/1 | 1/1 | 0 (0, 0) | 0 (0, 0) | 1 (1.6, 100);  **K103N**, **L100I^%^** | 0 (0, 0) | 1/1 | LA-I CAB+RPV | 1/1 (100) |
| 1. Soffritti et al., 2024 | Italy | 10.4 (median) | Q2M | 2 VL>20 c/mL or VL>200 c/mL | 68 | 28/74 (37.8)^¥^ | 68 | 2 (2.9) | 1/2;  1) UA  2) None | 2/2 | 0 (0, 0) | 2 (2.9, 100);  1) **G140S**, **Q148H**  2) **Q148R** | 2 (2.9, 100);  1) **181I**, **190A**  2) **138K** | 2 (2.9, 100) | 2/2 | PI, Multi-tablet;  1) DTG/RPV/MVC  2) DRV/c/FTC/TAF | 1/1 (100)  1) Y  2) UA |
| 1. Di Biagio & Gaggero, 2024 | Italy | 6 | Q2M | NR | 66 | NR | 66 | 1 (1.5) |  |  |  |  |  |  |  |  |  |
| 1. Mazzitelli et al., 2023 | Italy | 3 | Q2M | NR | 65 | 17 (26.2) | 65 | 1 (1.5) | 0/1 | 1/1 | 1 (1.5, 100) | 0 (0, 0) | 0 (0, 0) | 0 (0, 0) |  |  |  |
| 1. Yared et al., 2024 | USA | 12 | NR | 2 VL ≥ 30 c/mL at least 4 weeks apart | 58 | 10 (17.2) | 58 | 3 (5.2) | 1/3;  1) UA  2) UA  3) G140R | 3/3 | 0 (0, 0) | 3 (5.2, 100);  1) **E92K,** **Q146R**  2) **Q148K,** **E138K**  3) **Q148R** | 3 (5.2, 100);  1) **E138K**, **M230I**  2) **Y188L, V106I**  3) **E138K** | 3 (5.2, 100) |  |  |  |
| 1. Tincati et al., 2024 | Italy | 12 | Q2M | NR | 57 | 6 (10.5) | 57 | 0 (0) |  |  |  |  |  |  |  |  |  |
| 1. Iannone et al., 2024 | Italy | 12 | Q2M | NR | 53 | 12/74 (16.2)^¥^ | 53 | 1 (1.9) | 0/1 | 1/1 | 0 (0, 0) | 1 (1.9, 100);  **H51Y** | 0 (0, 0) | 0 (0, 0) | 1/1 | PI;  TAF/FTC/DRV/c | NR |
| 1. Roberts et al., 2023 | UK | NR | Q2M | NR | 51 | 1/33 (3.0)^¥^ | 33 | 0 (0) |  |  |  |  |  |  |  |  |  |
| 1. Ogilvy et al., 2024 | USA | 2 (minimum) | NR | VL>200 c/mL | 50 | 7/51 (13.7)^¥^ | 50 | 0 (0) |  |  |  |  |  |  |  |  |  |
| 1. Vega et al., 2024 | USA | 2 (minimum) | NR | VL ≥200 c/mL | 50 | 8/73 (11.1)^¥^ | 50 | 0 (0) |  |  |  |  |  |  |  |  |  |
| 1. Bowden et al., 2024 | Australia | 11 (median) | NR | VL ≥200 c/mL | 42 | 4/46 (8.7)^¥^ | 42 | 0 (0) |  |  |  |  |  |  |  |  |  |
| 1. Bobbio et al., 2024 | Italy | 3 | Q2M | NR | 42 | 7 (16.7) | 37 | 0 (0) |  |  |  |  |  |  |  |  |  |
| 1. Schiaroli et al., 2024 | Italy | 6 | Q2M | VL≥20 c/mL | 41 | 5/42 (11.9)^¥^ | 31 | 0 (0) |  |  |  |  |  |  |  |  |  |
| 1. Rutstein et al., 2024 | USA | 5 (median) | Q1M & Q2M | VL ≥50 c/mL | 41 | 11/47 (23.4)^¥^ | 32 | 1 (3.1) |  |  |  |  |  |  |  |  |  |
| 1. Koutsoupias et al., 2024 | USA | NR | NR | VL>201 c/mL | 39 | 17/42 (40.5)^¥^ | 39 | 0 (0) |  |  |  |  |  |  |  |  |  |
| 1. Konishi et al., 2024 | Japan | 11 | Q2M | ≥ 2 VL >200 c/mL | 38 | 0 (0) | 38 | 0 (0) |  |  |  |  |  |  |  |  |  |
| 1. Carraro et al., 2024 | Italy | 10.25 (median) | Q2M | NR | 36 | 11 (30.6) | 36 | 0 (0) |  |  |  |  |  |  |  |  |  |
| 1. Rigamonti et al., 2024 | Italy | 3 (minimum) | Q2M | NR | 34 | NR | 34 | 0 (0) |  |  |  |  |  |  |  |  |  |
| 1. Kirk et al., 2024 | USA | 12 | Q2M | VL≥200 c/mL | 33 | 9 (27.3) | 33 | 1 (3) | 1/1;  K103N | 1/1 | 0 (0, 0) | 1 (3, 100);  **E138A**, **G140S**, **Q148H**, **N155H** | 1 (3, 100);  **L100I**, K103N | 1 (3, 100) | 1/1 | PI;  DRV/c/FTR/DOR | 1/1 (100) |
| 1. Wijesinghe et al., 2023 | Canada | NR | NR | VL≥200 c/mL | 32 | 4 (12.5) | 32 | 0 (0) |  |  |  |  |  |  |  |  |  |
| 1. Fernández et al., 2024 (CAR-GR) | Spain | 4 | Q2M | VL≥20 c/mL | 31 | 15 (48.4) | 31 | 0 (0) |  |  |  |  |  |  |  |  |  |
| 1. Montalvo et al., 2023 | USA | 0.25 (minimum) | Q2M | VL ≥50 c/mL | 30 | 10 (33.3) | 29 | 0 (0) |  |  |  |  |  |  |  |  |  |
| 1. Williams et al., 2024 | USA | 33 | Q1M & Q2M | VL≥20 c/ml | 25 | 12 (48.0) | 25 | 0 (0) |  |  |  |  |  |  |  |  |  |
| 1. Masich et al., 2023 | USA | 12 | NR | VL >200 c/mL | 24 | NR | 24 | 2 (8.3) | 1/2;  1) None  2) UA | 2/2^#^ | 2 (8.3, 100) | 0 (0, 0) | 0 (0, 0) | 0 (0, 0) | 2/2 | INI, LA-I CAB+RPV  1) BIC/TAF/FTC  2) LA-I CAB+RPV | 1/1 (100)  1) UA  2) Y |
| 1. Ali et al., 2023 | UK | 9.75 (maximum) | Q2M | NR | 18 | NR | 18 | 0 (0) |  |  |  |  |  |  |  |  |  |
| 1. Chan et al., 2023 | China | 5 (median) | Q2M | NR | 18 | 1 (5.6) | 18 | 0 (0) |  |  |  |  |  |  |  |  |  |
| 1. Holland et al., 2023 | UK | 8 | Q2M | NR | 15 | 3 (20.0) | 15 | 0 (0) |  |  |  |  |  |  |  |  |  |
| 1. Spampinato et al., 2023 | Italy | 2 | Q2M | NR | 14 | 1 (7.1) | 14 | 0 (0) |  |  |  |  |  |  |  |  |  |
| 1. Perez et al., 2024 | USA | NR | Q2M | VL ≥200 c/ml | 11 | 3/15 (20.0)^¥^ | 11 | 0 (0) |  |  |  |  |  |  |  |  |  |

NR: Not reported, Q1M: monthly, Q2M: 2-monthly, VL: Viral load, VF: Viral failure, FU: Follow-up, INI: Integrase Inhibitor, LA-I CAB+RPV: long-acting cabotegravir and rilpivirine, N: No, NNRTI: Non-Nucleoside Reverse Transcriptase Inhibitor, PI: Protease Inhibitor, UA: Unavailable, Y: Yes, RAMs: resistance-associated mutations, UA: Unavailable, RNA: Ribonucleic acid, DNA: Deoxyribonucleic acid

^@^We converted durations to months and reported them as standard timepoints unless otherwise specified in parentheses (when no standard timepoint was provided); ^‡^Virally suppressed individuals included in the analysis; *We considered female individuals as those reported in observational cohort studies as any of the following: female gender or sex, cis-gender woman, or if only a male category was provided, we subtracted this from the total number. The percentage was calculated using the number of virally suppressed individuals at baseline as the denominator, unless a different denominator is indicated in the data cell; ^☥^ VF rates were calculated using the total number of virally suppressed individuals at risk of VF as the denominator; ^&^RAMs not listed under INI or NNRTI in the Stanford Algorithm were excluded. Bolded RAMs were not present in baseline/historical genotypes prior to VF. n/N RAMs rate was calculated using the total number of virally-suppressed individuals at risk of VF as the denominator; ^ⵜ^Based on definition of suppression or individuals reported as ‘resuppressed’ by individual studies;^£^Baseline resistance testing only available for reverse transcriptase RAMs; ^%^These RAMs were not detected before VF; ∞Historical Genotype only available for NNRTI RAMs; ^@@^None had RPV DRMs at baseline; ^¥^ The denominator differs from the total number of virally suppressed individuals at baseline because gender was not reported discretely for this population (e.g., data provided includes individuals non-virally suppressed at switch or describes the referral or analysis population); ^©^When multiple post-VF regimens were listed, we reported the one provided at the moment of re-suppression (if data available), or otherwise the first regimen listed; ^Absence of NNRTI RAMs at baseline may be inferred due to country-specific eligibility requirements for LA-I CAB+RPV; ^✖^ Data not matched to specific VFs; ^#^Baseline or historic RAMs were excluded from genotype results at VF; ^$^Data was also extracted from: *van Welzen BJ, et al. Virological failure after switch to long-acting cabotegravir and rilpivirine injectable therapy: An in-depth analysis. Clin Infect Dis. 2024;79(1):189-95.*

# Full reference list of observational cohort studies included in the systematic review

Adachi, E., Saito, M., Otani, A., Koga, M., & Yotsuyanagi, H. (2024). Brief communications: changes in inflammatory biomarkers and lipid profiles after switching to long-acting cabotegravir plus rilpivirine. *Aids Research and Therapy*, *21*(1), 1. <https://doi.org/10.1186/s12981-023-00590-4>

Ali, B., Nye, C., Oakley, R., Clark, F., George, T., & Underwood, J. (2023). TP04. An innovative, patient-centred approach to delivery of long-acting injectable antiretroviral therapy to people living with HIV using pre-existing outpatient parenteral antimicrobial therapy (OPAT) services. *HIV Medicine*, *24*(S3), 18-19. <https://doi.org/https://doi.org/10.1111/hiv.13477>

Antonucci, F., Santoro, M. M., Bertoli, A., Torre, G., Ferrara, S., Grillo, C., Narducci, A., Ceccherini-Silberstein, F., Santantonio, T., & Caputo, S. L. (June 19-21, 2024). Efficacy and tolerability of long-acting cabotegravir + rilpivirine in real-world setting, 52 weeks results. Italian Conference on AIDS and Antiviral Research, Rome, Italy.

Bhayani, N. K., Bernett, J. R., Sleweon, T. K., Young, E. M., Stock, K. J., Baker, S. E., Luu, Q., Metzger, B. S., Couch, K. A., Weeks, C. J., & Anglen, L. J. V. (October 16-19, 2024). Infectious Disease Physician Office-Based HIV Program Results in Successful Adherence of Therapy in Patients Receiving Long-Acting Cabotegravir and Rilpivirine. IDWeek, Los Angeles, California

Biagio, A. D., & Gaggero, E. (June 19-21, 2024). Description of a cohort of people with HIV infection on long-acting antiretroviral therapy. Italian Conference on AIDS and Antiviral Research, Rome, Italy.

Bobbio, N., Fiorellino, D., Puppo, S., Vacca, E. B., Boni, S., Puente, F. D., Feasi, M., Parisini, A., Prinapori, S. R., Tigano, & Pontali, E. (June 19-21, 2024). Experience with the use of prolonged-release injectable CAB/RPV in outpatients. Italian Conference on AIDS and Antiviral Research, Rome, Italy.

Bowden, B., Lim, Y., & Acklom, K. (September 15-17, 2024). Long Acting Cabotegravir/Rilpivirine Uptake, Adherence and Discontinuation in a High Case Load Publicly Funded Sexual Health Service in Central Sydney, New South Wales. The Australasian HIV & AIDS Conference, Sydney, Australia.

Canavesi, G., Mena, M., Zaninetta, E., Gazzola, L., Bini, T., Bo, G., Diaz, D. A., Orofino, G., Vito, A. D., Madeddu, G., Grillo, C., Bartalucci, C., Centorrino, F., Squillace, N., Bonfanti, P., Rapino, S., Tiecco, G., Focà, E., Menozzi, M., . . . Rusconi, S. (November 10-13, 2024). Cabotegravir-rilpivirine long-acting injectable regimen: an analysis of the causes of interruption and impact of genotypic drug resistance in a multicentric cohort. HIV Drug Therapy Glasgow, Glasgow, Scotland.

Cantor, A., Vaill, R., & Radix, A. (July 20-26, 2024). Challenges and Progress in Implementing Long-Acting Antiretroviral Therapy at an Urban Community Health Center. 25th International AIDS Conference, Munich, Germany.

Carraro, A., Marocco, R., Mancarella, G., Zingaropoli, A., Tortellini, E., Guardiani, S., Maria, S. D., Corazza, S., Grimaldi, A., Gasperin, A., Zuccalà, P., Parente, A., Ansaldo, L., D'Achille, M., Rossi, V., D'Onofrio, O., Addio, P., Borgo, C. D., & Lichtner, M. (June 19-21, 2024). Preliminary real world experience in a cohort of PLWH undergoing LA CAB/RPV in Latina: a heterogeneous population with homogeneous efficacy and satisfaction. Italian Conference on AIDS and Antiviral Research, Rome, Italy.

Chan, J., Chik, S., Lau, P., Choi, Y., Lau, W., Leung, C., To, P., Law, T., Lamb, S., & Tsang, O. (October 18-21, 2023). Real world use of long-acting cabotegravir and rilpivirine in a HIV centre in Hong Kong. 19th European AIDS Conference, Warsaw, Poland.

Chiu, C. A., Mulligan, N., Weng, B., Zakhary, B., La, M., & Wu, P. (October 16-19, 2024). Real-World Experience with Long-Acting Intramuscular Cabotegravir and Rilpivirine in Adults for the Maintenance of HIV-1 Suppression in County-Based Clinics in Riverside County. IDWeek, Los Angeles, California

Dannenberg, C., Matthews, H., Almahfoud, M., Weimann, L., Scheiter, R. L., Hüfner, A.-D., Schmiedel, S., Jordan, S., Wiesch, J. S. z., & Degen, O. (November 10-13, 2024). Long-term treatment adherence and virological suppression in an outpatient cohort of people living with HIV (PLWH) on long-acting injectable HIV therapy. HIV Drug Therapy Glasgow, Glasgow, Scotland

Dawiec, M., Gasiorowski, J., Szymczak, A., Zinczuk, A., Inglot, M., Knysz, B., Zielinska, K., Furdal, M., & Szetela, B. (November 10-13, 2024). EFFICACY AND SAFETY OF LONG-ACTING CABOTEGRAVIR + RILPIVIRINE AT ALL SAINTS CLINIC IN WROCLAW (POLAND) –A 2-YEAR REAL-LIFE SINGLE-CENTER EXPERIENCE. HIV Drug Therapy Glasgow, Glasgow, Scotland.

Derrick, C. B., Magee, M., Tsai, Y. V., Pizzuti, M. E., Parker, S., Lucas, O., Taylor, K., Albright, R., Langehans, B., Schreiber, D., Guest, G., Ahuja, D., & Weissman, S. (2025). Process evaluation and early outcomes of real-world implementation of a pharmacist-driven cabotegravir/rilpivirine long-acting injectable initiative. *Am J Health Syst Pharm*, *82*(4), 144-149. <https://doi.org/10.1093/ajhp/zxae260>

Deschanvres, C., Allavena, C., Palich, R., Rey, D., Cheret, A., Zaegel-Faucher, O., & Hocqueloux, L. (October 18-21, 2023). Cabotegravir-Rilpivirine Long acting : Data in real life setting in a French Cohort. 19th European AIDS Conference, Warsaw, Poland.

Eron, J. J., Sarkar, S., Frick, A., Radtchenko, J., Sridhar, G., Ragone, L., Mounzer, K., Santiago, S., Wyk, J. v., Elion, R., & Vannappagari, V. (March 3-6, 2024). Real-World Utilization of Cabotegravir + Rilpivirine in the US: Data From Trio Health Cohort. Conference on Retroviruses and Opportunistic Infections, Denver, Colorado.

Fernández-Hinojal, F., Grela, A. D. G., Martin-Carbonero, L., Valencia, E., Mican, R., Busca, C., Ramos, L., Arribas, J., González-Garcia, J., Bernardino, J., & Montes, M. (October 18-21, 2023). Effectiveness and timing of viral load measurement in real-world use of Long-acting Cabotegravir-Rilpivirine in People with HIV. 19th European AIDS Conference, Warsaw, Poland.

Fernández, A., Scévola, S., Niubó, J., Sykes, C., Schauer, A. P., Piatti, C., Morenilla, S., Sedó, A., Soriano, I., García, B., Medina, D., Tiraboschi, J., Saumoy, M., Cottrell, M. L., & Imaz, A. (November 10-13, 2024). Cabotegravir and Rilpivirine concentrations and HIV-1 RNA suppression in male and female genital fluids and rectal tissue in people with HIV on antiretroviral therapy with long-acting intramuscular Cabotegravir plus Rilpivirine. HIV Drug Therapy Glasgow, Glasgow, Scotland.

Ferrara, M. (June 19-21, 2024). Long Acting Cabotegravir and Rilpivirine plasma and intracellular Pharmacokinetics in the Clinical Setting. Italian Conference on AIDS and Antiviral Research, Rome, Italy.

Fessler, D., Kelley, E., McLaughlin, R., Loubier, E., Bangert, B., Adams, T., Fuchs, A., Alves, L., Estrada, J., & Henn, S. (March 3-6, 2024). Long-Acting ART in a Community Health Center: Insights and Early Outcomes. Conference on Retroviruses and Opportunistic Infections, Denver, Colorado.

Gagliardini, R., Benedittis, S. D., Tavelli, A., Lapadula, G., Mazzotta, V., Bruzzesi, E., Cervo, A., Carrozzo, G., Saracino, A., Rusconi, S., Marchetti, G., Ceccherini-Silberstein, F., Antinori, A., Monforte, A. d. A., & Muccini, C. (November 10-13, 2024). EFFECTIVENESS OF LONG-ACTING ART WITH CABOTEGRAVIR/RILPIVIRINE IN THE ICONA COHORT. HIV Drug Therapy Glasgow, Scotland.

Gandhi, M., Hickey, M., Imbert, E., Grochowski, J., Mayorga-Munoz, F., Szumowski, J. D., Oskarsson, J., Shiels, M., Sauceda, J., Salazar, J., Dilworth, S., Nguyen, J. Q., Glidden, D. V., Havlir, D. V., & Christopoulos, K. A. (2023). Demonstration Project of Long-Acting Antiretroviral Therapy in a Diverse Population of People With HIV. *Ann Intern Med*, *176*(7), 969-974. <https://doi.org/10.7326/m23-0788>

González-Cordón, A., Inciarte, A., Mosquera, M., Berrocal, L., Martínez-Rebollar, M., Torres, B., Chivite, I., Arreba, P., Ambrosioni, J., Foncillas, A., Mora, L. d. l., Calvo, J., Sempere, A., Miró, J. M., Llobet, R., Lazzari, E. d., Mallolas, J., Martínez, E., Laguno, M., & Blanco, J. L. (November 10-13, 2024). Prevalence of virological failure and resistance patterns to Long-Acting Cabotegravir-Rilpivirine: A real life single center cohort study. HIV Drug Therapy Glasgow, Glasgow, Scotland

Gutiérrez, F., Fernández-González, M., Ledesma, C., Losada-Echeberría, M., Barrajón-Catalán, E., García-Abellán, J., De Stefano, D., López, L., Bello-Perez, M., Padilla, S., & Masiá, M. (2024). Virological History Predicts Non-sustained Viral Suppression With Long-Acting Cabotegravir and Rilpivirine Therapy, Independent of Pharmacokinetic Parameters. *Clinical Infectious Diseases*. <https://doi.org/10.1093/cid/ciae475>

Haser, G. C., Balter, L., Gurley, S., Thomas, M., Murphy, T., Sumitani, J., Leue, E. P., Hollman, A., Karneh, M., Wray, L., Washington, M., Corbin-Johnson, D., Condra, A., Niles-Carnes, L., Smith, B. L., Armstrong, W. S., Kalokhe, A. S., Colasanti, J. A., & Collins, L. F. (2024). Early Implementation and Outcomes Among People with HIV Who Accessed Long-Acting Injectable Cabotegravir/Rilpivirine at Two Ryan White Clinics in the U.S. South. *AIDS Research and Human Retroviruses*, *40*(12), 690-700. <https://doi.org/10.1089/aid.2024.0007>

Hessamfar, M., Leleux, O., Mazeau, A. P., Krzyzanowsky, C., Moal, G. L., Neau, D., Ferrand, H., Desclaux, A., Lazaro, E., Duffau, P., Gérard, Y., Vandenhende, M.-A., & Bonnet, F. (November 10-13, 2024). Use of long acting Cabotegravir and Rilpivirine in a real life setting: 12 month results of virological outcome, adherence, safety, durability, in the ANRS CO3 AquiVIH NA Cohort France. HIV Drug Therapy Glasgow, Glasgow, Scotland.

Hidalgo-Tenorio, C., Aguilera, M. Á., Santos, I. D. L., Rivero, A., Lirola, A. L., Lopez, T., Ruiz, A., Romero, A., Bernal, E., Sorni, P., Moreno, S., Omar, M., Sanjoaquin, I., Martinez, O., & Garcia, C. (November 10-13, 2024). Results at month 7 of CABO CHANCE study: real world evidence (RWE) on the use of intramuscular cabotegravir plus rilpivirine lon g acting (CAB+RPV LA) dosed every two months in virally suppressed people with HIV (PWH). HIV Drug Therapy Glasgow, Glasgow, Scotland.

Hill, L., Yin, J., Patel, N., Abulhosn, K., Suarez, E., Karim, A., & Bamford, L. (2025). Predictors of Injection Visit Adherence in Those Receiving Injectable Cabotegravir/Rilpivirine. *J Acquir Immune Defic Syndr*, *98*(2), 185-192. <https://doi.org/10.1097/qai.0000000000003556>

Holland, K., Anjum, R., Quinn, G., Arumainayagam, J., & Acharya, S. (2023). P006. Successful implementation of a novel treatment strategy: challenges, outcomes and patient perspectives. *HIV Medicine*, *24*(S3), 24-25. <https://doi.org/https://doi.org/10.1111/hiv.13478>

Hsu, R. K., Sension, M., Fusco, J. S., Brunet, L., Cochran, Q., Sridhar, G., Vannappagari, V., Wyk, J. V., Wohlfeiler, M., Levis, B., & Fusco, G. P. (March 3-6, 2024). Real-World Effectiveness of Cabotegravir + Rilpivirine vs Standard of Care Oral Regimens in the US. Conference on Retroviruses and Opportunistic Infections Denver, Colorado.

Iannone, V., Rossotti, R., Bana, N. B., Cavazza, G., D’Amico, F., Lombardi, F., Salvo, P. F., Baldin, G., Giambenedetto, S. D., Bernacchia, D., Pagani, G., Borghetti, A., & Rusconi, S. (June 19-21, 2024). Unconventional use of Long-Acting CAB+RPV against HIV in PWH in need: real-world data at 48 weeks from an Italian bicentric cohort. Italian Conference on AIDS and Antiviral Research, Rome, Italy.

Jonsson-Oldenbüttel, C., Noe, S., Wyen, C., Borch, J., Ummard-Berger, K., Postel, N., Scholten, S., Dymek, K. M., Westermayer, B., Rios, P. d. l., & Scherzer, J. (22-26 July, 2024). 12-Month Outcomes of Cabotegravir Plus Rilpivirine Long-Acting Every 2 Months in a Real-World Setting: Effectiveness, Adherence to Injections, and Patient-Reported Outcomes From People With HIV-1 in the German CARLOS Study. 25th International AIDS Conference, Munich, Germany.

Kirk, S. E., Young, C., Berry, H., Hanson, R., Moreland, A., Fonner, V., Gebregziabher, M., Williams, J., & Meissner, E. G. (2024). Comparison of At-Home Versus In-Clinic Receipt of Long-Acting Injectable Cabotegravir/Rilpivirine. *Clinical Infectious Diseases*. <https://doi.org/10.1093/cid/ciae472>

Konishi, K., Onozuka, D., Okubo, M., Kasamatsu, Y., Kutsuna, S., & Shirano, M. (2024). Long-acting antiretroviral therapy effectiveness and patient satisfaction using patient questionnaires: data from a real-world setting. *Bmc Infectious Diseases*, *24*(1), 979. <https://doi.org/10.1186/s12879-024-09904-x>

Koutsoupias, P., French, K., & Wortmann, G. (October 16-19, 2024). Analysis of Cabotegravir-Rilpivirine Use in HIV patients at MedStar Washington Hospital Center. IDWeek, Los Angeles, California

Lagi, F., Formica, G., Fabbiani, M., Rossetti, B., Piccica, M., Giachè, S., Messeri, D., Costarelli, S., Riguccini, E., Sarteschi, G., Gennaro, M. D., Francalanci, E., Gasparro, G., Fognani, M., Paggi, R., Corsi, P., Pozzi, M., Sterrantino, G., Tumbarello, M., . . . Bartoloni, A. (November 10-13, 2024). DISCONTINUATION OF RILPIVIRINE AND CABOTEGRAVIR IN HIV-1 VIROLOGICALLY SUPPRESSED ADULTS: A MULTICENTER OBSERVATIONAL STUDY IN TUSCANY (LAHIV STUDY). HIV Drug Therapy Glasgow, Glasgow, Scotland.

Liegeon, G., Kaperak, C., Friedman, E., Dawdani, A., Djuricich, P., Stafford, K., Schmitt, J., Hazra, A., Christopoulos, K., Schneider, J., & McNulty, M. (March 3-6, 2024). Injectable Cabotegravir/Rilpivirine Reach and Effectiveness in a South Side Chicago HIV Clinic. Conference on Retroviruses and Opportunistic Infections, Denver, Colorado.

Liu, Y., Fisk-Hoffman, R., Patel, M., Cook, R., & Prosperi, M. (March 3-6, 2024). Uptake of long-acting injectable antiretroviral therapy in Florida: An assessment of EHR data 31st Conference on Retroviruses and Opportunistic Infections Colorado, USA.

Maguire, C., Rueve, K., Farmer, E., Huesgen, E., Karaj, A., Binkley, A., Mounzer, K., Brizzi, M., Chary, P., Sung, P., Graziani, A., Hiserodt, E., Baron, J., Koenig, H., & Short, W. R. (2024). Real world virologic outcomes in patients with elevated body mass index receiving long acting cabotegravir/rilpivirine. *Clin Infect Dis*. <https://doi.org/10.1093/cid/ciae579>

Martín, L. B., Montes, M. L., Puerto, M. J. G., Torralba, M., Pousada, G., Santacreu, M., Santos, I. d. l., Úbeda, A. C., Clotet, N. C., Canales, M. J. C., Amado, L. E. M., Rico, P. M., Hernández, C. M., Santiago, A. D. d., Cecilio, Á., Fernández, M. Z., Morell, E. B., Sepúlveda, M. A., Gallego, M. J. V., . . . Troya, J. (November 10-13, 2024). REAL WORLD OUTCOMES OF CABOTEGRAVIR AND RILPIVIRINE FOR TREATING PLHIV IN SPAIN: A MULTICENTRE, AMBISPECTIVE AND NATIONWIDE STUDY (THE RELATIVITY COHORT). HIV Drug Therapy Glasgow, Glasgow, Scotland.

Masich, A. M., Gomes, D., Higginson, R. T., Morgan, Z., Nixon, D., Tran, M., Winthrop, E., & Fulco, P. P. (2023). HIV virologic response and baseline genotypic resistance in a long-acting cabotegravir/rilpivirine initiation program. *AIDS*, *37*(10), 1641-1642. <https://doi.org/10.1097/qad.0000000000003590>

Matone, M., Piscaglia, M., Giacomelli, A., Moschese, D., Capetti, A., Pozza, G., Galli, L., Antinori, S., Gori, A., Rizzardini, G., & Cossu, M. V. (2024). Brief Report: Switching to Long-Acting CAB/RPV: Data From an Italian Monocentric Cohort. *JAIDS Journal of Acquired Immune Deficiency Syndromes*, *97*(4), e1-e5. <https://doi.org/10.1097/qai.0000000000003501>

Mazzitelli, M., Agostini, E., Sasset, L., Leoni, D., Gardin, S., Presa, N., Putaggio, C., Bragato, B., Parisi, S., & Cattelan, A. (October 18-21, 2023). Real life data on clinical and laboratory outcomes of long acting cabotegravir and rilpivirine in people with HIV. 19th European AIDS Conference, Warsaw, Poland.

Mesa, D., Ryscavage, P., Schmalzle, S., Seung, H., & Pandit, N. S. (October 11-15, 2023). Real-world Outcomes with Cabotegravir/Rilpivirine: Does Duration of Pre-treatment Viral Suppression Matter? IDWeek, Boston, Massachusetts.

Montalvo, S., Sherman, E., Eckardt, P. A., Bromberg, R., Poon, K. K., Ostran, G. V., Cevallos, E. C., & Savage, A. (October 11-15, 2023). (1591) Real-world experiences and outomes implementing long-acting cabotegravir/rilpivirine at a Ryan White HIV/AIDS Program (RWHAP)-funded clinic in South Florida. IDWeek, Boston, Massachusetts.

Muccini, C., Gianotti, N., Diotallevi, S., Lolatto, R., Spagnuolo, V., Canetti, D., Bagaglio, S., Perez, V. G., Clemente, T., Bottanelli, M., Candela, C., Nozza, S., & Castagna, A. (2024). One Year of Long-Acting Cabotegravir and Rilpivirine in People With Human Immunodeficiency Virus and Long Exposure to Antiretroviral Therapy: Data From the SCohoLART Study. *Open Forum Infectious Diseases*, *11*(7). <https://doi.org/10.1093/ofid/ofae326>

Nasser, K., Valentin, A.-M., & Ramgopal, M. (October 18-21, 2023, October 18-21, 2023). Real-world clinical outcomes of HIV-1 virologically suppressed adults on bictegravir/emtricitabine/tenofovir alafenamide who switched to long-acting intramuscular cabotegravir+rilpivirine at 12-, 24-, and 48-weeks 19th European AIDS Conference, Warsaw, Poland.

Nguyen, N. M., Kavanagh, R., Gozar, M., Cabral, D., Goetz, H., Cha, A., McGowan, J. P., & Pao, M. L. (2024). Implementation of a Pharmacist-Led, Long-Acting, Injectable Cabotegravir/Rilpivirine Program for HIV-1 at Health System-Based Clinics in the New York Metropolitan Area. *AIDS Patient Care STDS*, *38*(3), 115-122. <https://doi.org/10.1089/apc.2023.0250>

Nunnari, G., Spampinato, S., Conti, G. N., Giarratana, C., Cirelli, T., Coco, V., Raddusa, M. S. P., Fisicaro, V., Gullotta, C., Bruno, R., Pistarà, E., Villari, N., BrunoCacopardo, Rullo, E. V., Marino, A., Celesia, B. M., Pellicanò, G., & Mirabile, A. (2024). P090. PLWH treated with cabotegravir-rilpivirine have increasedCD4/CD8 ratio and decreased CD8 T cells, as expressionof lower inflammation, while maintaining virological suppression: a real-life 48 weeks analysis. *Journal of the International AIDS Society*, *27*(S6), 100. <https://doi.org/https://doi.org/10.1002/jia2.26370>

Ogilvy, A., Ware, C., Lucar, J., Ruiz, M., Roberts, A., Siegel, M., & Semeniuk, O. (October 16-19, 2024). Real-World Experience with Long-acting Injectable Cabotegravir/Rilpivirine (CAB/RPV LA) In an Academic Medical Center

IDWeek, Los Angeles, California

Palacios, R., Gómez-Ayerbe, C., Mayorga, M., Espinosa, N., Hidalgo, A., Téllez, F., Lozano, A. B., Loring, M., & Santos, J. (November 10-13, 2024). Real-World Utilization of Cabotegravir + Rilpivirine in Southern Spain: Data From the CARIPLA Study. HIV Drug Therapy Glasgow, Glasgow, Scotland.

Perez, A., Nieves, S., & Meisner, J. (2024). Implementation of Injectable Cabotegravir/Rilpivirine for Treatment of Human Immunodeficiency Virus in Patients With Substance Use Disorders at a Syringe Exchange Clinic. *Open Forum Infectious Diseases*, *11*(11). <https://doi.org/10.1093/ofid/ofae640>

Pérez, S., Petersen, E. A., & Huhn, G. (October 11-15, 2023). Real-World Efficacy of Long-Acting Cabotegravir and Rilpivirine in an Urban HIV Clinic IDWeek, Boston, Massachusetts.

Pozniak, A., Sridhar, G., Assoumou, L., Piroth, L., Braun, D., Fletcher, C., Ragone, L., Shah, A., Wyk, J. v., & Vannappagari, V. (July 20-26, 2024). Real-world utilization and effectiveness of long-acting cabotegravir + rilpivirine in virologically suppressed treatment experienced individuals in Europe: Data from COMBINE-2 Cohort Study. 25th International AIDS Conference, Munich, Germany.

Prather, C., Richey, K., & Verderese, J. P. (October 16-19, 2024). A Descriptive Report of Discontinuation Rates, Blips, and Viremia Associated with Cabotegravir/Rilpivirine at One Ryan White HIV/AIDS Program-funded Site in Northern Virginia. IDWeek, Los Angeles, California

Psomas, K., Halfon, P., Salnikova, M., Khiri, H., Tichadelle, F., Allemand, J., Néant, N., Solas, C., & Philibert, P. (October 18-21, 2023). Clinical and pharmacological outcomes of real-world use of Long-Acting Cabotegravir and Rilpivirine in France; efficacy and tolerance during the first 72 weeks. 19th European AIDS Conference, Warsaw, Poland.

Ramirez, J. D. (October 18-21, 2023). Uptake and Discontinuation of the Long-Acting Duo in the Swiss HIV Cohort Study: Preliminary Analysis on Cabotegravir + Rilpivirine. 19th European AIDS Conference, Warsaw, Poland.

Ribera, A. F., Santos, P. M., Gil, A. H., Pia, C. P., Sempere, R. O., Domingo, A. B., Gomez, S. P., & Puerto, M. J. G. (November 26-29, 2023). Tratamiento con Cabotegravir/Rilpivirina administrado cada dos meses en vida real: experiencia de un hospital terciario. XIV edición del Congreso de GESIDA, Coruña, Spain.

Rigamonti, C., Marzolla, D., Giglia, M., Cretella, S., Vitale, S., & Calza, L. (June 19-21, 2024). Exploring effectiveness and tolerance: switching to long-acting cabotegravir plus rilpivirine therapy in virologically suppressed individuals living with HIV. Italian Conference on AIDS and Antiviral Research, Rome, Italy.

Ring, K., Smuk, M., Shongwe, M., Okonta, L., Mackie, N. E., Ayres, S., Barber, T. J., Akodu, J., Ferro, F., Chilton, D., Hurn, E., Halai, B., Barchi, W., Ali, A., Darko, S., White, G., Clarke, E., Clark, F., Ali, B., . . . Orkin, C. (2024). Multicentre service evaluation of injectable cabotegravir and rilpivirine delivery and outcomes across 12 UK clinics (SHARE LAI-net). *HIV Medicine*, *25*(10), 1125-1134. <https://doi.org/https://doi.org/10.1111/hiv.13679>

Roberts, J., Richardson, C., Jaquiss, J., Clarke, A., & Paul, L. (October 18-21, 2023). A shot in the dark: a review one year on after initiation of injectable HIV Treatment in a UK HIV outpatient clinic. 19th European AIDS Conference, Warsaw, Poland.

Rubenstein, E., Diemer, M., Goldwirt, L., Lascoux, C., Lafaurie, M., Ponscarme, D., Denis, B., Castro, N. D., Rami, A., Sellier, P.-O., Deville, L., Chaix, M.-L., Delaugerre, C., & Molina, J.-M. (October 18-21, 2023). Low cabotegravir trough concentrations without oral lead-in in patients with HIV-1 infection switching to long-acting cabotegravir and rilpivirine. 19th European AIDS Conference, Warsaw, Poland.

Rutstein, S. E., Lopez, C., Davy-Mendez, T., Agarwal, H., Huffstetler, H., Perhac, A., Turner, B., Eron, J. J., Go, V., Farel, C. E., Li, K.-P., & Napravnik, S. (2024). Characterizing long-acting injectable antiretroviral therapy eligibility and initiation at a safety net academic medical center in the southeastern United States. *International Journal of STD & AIDS*, *36*(1), 47-55. <https://doi.org/10.1177/09564624241289998>

Schiaroli, E., Tordi, S., Svizzeretto, E., Tommasi, A., Zoffoli, A., & Francisci, D. (June 19-21, 2024). The Long Acting Therapy with CAB/RPV: experience at the Infectious Diseases Clinic of Perugia. Italian Conference on AIDS and Antiviral Research, Rome, Italy.

Schneider, S., Sension, M., Dretler, A., Lalla-Reddy, S., Schubert, C., Merrill, D., Richardson, D., Sherif, B., Zografos, L., & Garris, C. (July 20-26, 2024). Clinical Outcomes at Month 12 After Initiation of Cabotegravir and Rilpivirine Long-Acting (CAB+RPV LA) in an Observational Real-World Study (BEYOND). 25th International AIDS Conference, Munich, Germany.

Seang, S., Palacios, C., Valin, N., Gansou, A., Faycal, A., Vincensini, J.-P., Abdi, B., Lambert-Niclos, S., Peytavin, G., Katlama, C., Pialoux, G., Lacombe, K., Pourcher, V., & Valantin, M.-A. (October 18-21, 2023). HIV viremias among patients who discontinued Cabotegravir (CAB)/Rilpivirine (RPV) Long acting (LA) Intramuscular (IM) in real-life setting. 19th European AIDS Conference, Warsaw, Poland.

Serris, A., Ferre, V. M., Le Hingrat, Q., Bachelard, A., Charpentier, C., Exarchopoulos, M., Damond, F., Phung, B. C., Landman, R., Yazdanpanah, Y., Descamps, D., Joly, V., Peytavin, G., & Ghosn, J. (2024). Real-world data on long-acting intramuscular maintenance therapy with cabotegravir and rilpivirine mirror Phase 3 results. *J Antimicrob Chemother*, *79*(11), 2932-2938. <https://doi.org/10.1093/jac/dkae308>

Shankaran, S., Hernandez-Guarin, L., Jhobalia, N., Sha, B., & Aziz, M. (March 3-6, 2024). Virologic Failure with Cabotegravir-RilpivirineInjections: A Single-Site Experience. Conference on Retroviruses and Opportunistic Infections, Denver, Colorado.

Soffritti, A., Menozzi, M., Cervo, A., & Mussini, C. (June 19-21, 2024). Long-Acting injectable regimen with cabotegravir + rilpivirine in people living with HIV: real-life experience from Modena HIV. Italian Conference on AIDS and Antiviral Research, Rome, Italy.

Spampinato, S., Saia, A. G., Russotto, Y., Micali, C., Ceccarelli, M., Rullo, E. V., Pellicanò, G. F., & Nunnari, G. (June 14-16, 2023). Real Life efficacy and satisfaction of long-acting ART Cabotegravir-Rilpivirine: new era of ART. Italian Conference on AIDS and Antiviral Research, Bari, Italy.

Taramasso, L., Squillace, N., Ricci, E., Ferrara, S., Orofino, G., Sarchi, E., Pontali, E., Cenderello, G., Pellicanò, G. F., Lagi, F., Salomoni, E., Bargiacchi, O., Carleo, M. A., Pusterla, L., Martini, S., Bellagamba, R., Madeddu, G., Socio, G. V. D., Menzaghi, B., . . . Bonfanti, P. (November 10-13, 2024). Causes of discontinuation of long acting cabotegravir and rilpivirine in clinical practice. Results from the prospective multicenter SCOLTA cohort. HIV Drug Therapy Glasgow, Glasgow, Scotland.

Tincati, C., Rezzonico, L. F., Santoro, A., Ferrante, E., Bini, T., Gazzola, L., & Marchetti, G. (November 10-13, 2024). T-cell Homeostasis Parameters Following Switch to Injectable Cabotegravir plus Rilpivirine in Virally-Suppressed People Living with HIV (PLWH). HIV Drug Therapy Glasgow, Glasgow, Scotland.

Torralba, M., Alamo, M. R. D., Demetrio, A. C., Martinez, E., Lazaro, A., & Delgado, A. (November 26-29, 2023). PRIMERAS EXPERIENCIAS EN VIDA REAL CON EL USO DE CABOTEGRAVIR+RILPIVIRINA DE ACCION PROLONGADA TRAS SU RECIENTE COMERCIALIZACION. XIV edición del Congreso de GESIDA, Coruña, Spain.

Vega, V. A., Aragon, K. G., Azimi, S. F., Snyder, J. W., Iandiorio, M. J., & Jakeman, B. A. (October 16-19, 2024). Injecting Hope: A Long-Acting Injectable Program to Treat HIV Using Cabotegravir/Rilpivirine at a Ryan-White Funded HIV Clinic. IDWeek, Los Angeles, California

Wijesinghe, A., Yoong, D., Gough, K., & Tan, D. H. S. (April 27-30, 2023). 347 A Descriptive Analysis of Patients Receiving Injectable ART in a Toronto clinic. The 32nd Annual Canadian Conference on HIV / AIDS Research, Quebec City, Canada.

Williams, T., Anderson, L., Unternaher, J., Koay, W. L. A., Bright, K., Mareuil, J., & Rakhmanina, N. (22-26 July, 2024). Adolescents and Young Adults with HIV Using Long- Acting Injectable Cabotegravir /Rilpivirine as a Standard of Care: Outcomes of the Observational Cohort at 33 months. 25th International AIDS Conference, Munich, Germany.

Yared, N., Gudipati, S., Payne, S., & Brar, I. (October 16-19, 2024). Assessment of Failures of Long-Acting Cabotegravir and Rilpivirine in a Real-World Treatment Setting. IDWeek, Los Angeles, California

# Appendix References

1. Downs SH, Black N. The feasibility of creating a checklist for the assessment of the methodological quality both of randomised and non-randomised studies of health care interventions. J Epidemiol Community Health. 1998;52(6):377-84.

2. Shivakumar S, Srivastava A, G CS. Body Mass Index and Dental Caries: A Systematic Review. Int J Clin Pediatr Dent. 2018;11(3):228-32.

3. Canuto R, da Silva Garcez A, Kac G, de Lira PIC, Olinto MTA. Eating frequency and weight and body composition: a systematic review of observational studies. Public Health Nutr. 2017;20(12):2079-95.

4. Silva AE, Menezes AM, Demarco FF, Vargas-Ferreira F, Peres MA. Obesity and dental caries: systematic review. Rev Saude Publica. 2013;47(4):799-812.
